# Development of a PDEδ‐Targeting PROTACs that Impair Lipid Metabolism

**DOI:** 10.1002/anie.201913904

**Published:** 2020-01-22

**Authors:** Michael Winzker, Alexandra Friese, Uwe Koch, Petra Janning, Slava Ziegler, Herbert Waldmann

**Affiliations:** ^1^ Department of Chemical Biology Max-Planck-Institute of Molecular Physiology Otto-Hahn-Straße 11 44227 Dortmund Germany; ^2^ Lead Discovery Center GmbH Otto-Hahn-Straße 15 44227 Dortmund Germany; ^3^ Faculty of Chemistry and Chemical Biology Technical University Dortmund Otto-Hahn-Straße 6 44227 Dortmund Germany

**Keywords:** lipids, metabolism, PDEδ, proteolysis-targeting chimeras (PROTACs), proteomics

## Abstract

The prenyl‐protein chaperone PDEδ modulates the localization of lipidated proteins in the cell, but current knowledge about its biological function is limited. Small‐molecule inhibitors that target the PDEδ prenyl‐binding site have proven invaluable in the analysis of biological processes mediated by PDEδ, like KRas cellular trafficking. However, allosteric inhibitor release from PDEδ by the Arl2/3 GTPases limits their application. We describe the development of new proteolysis‐targeting chimeras (PROTACs) that efficiently and selectively reduce PDEδ levels in cells through induced proteasomal degradation. Application of the PDEδ PROTACs increased sterol regulatory element binding protein (SREBP)‐mediated gene expression of enzymes involved in lipid metabolism, which was accompanied by elevated levels of cholesterol precursors. This finding for the first time demonstrates that PDEδ function plays a role in the regulation of enzymes of the mevalonate pathway.

## Graphical abstract

**Right on target**: An analogue of deltasonamide 1, an inhibitor of the prenyl‐protein chaperone PDEδ, was employed for the design and synthesis of proteolysis‐targeting chimeras (PROTACs) based on ligands for the E3 ligases cereblon or VHL to target PDEδ for degradation. The PROTACs efficiently, selectively, and proteasome‐dependently deplete PDEδ in cells and hold promise as valuable tools for chemical PDEδ knockdown.

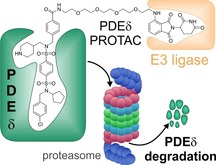

The prenyl‐binding protein PDEδ (retinal rod rhodopsin‐sensitive cGMP 3′,5′‐cyclic phosphodiesterase subunit delta; PDE6D or PrBP/δ)^1^, ^2^ binds, solubilizes, and thereby sustains the spatial organization of prenylated GTPases like Ras and Rheb^2^, ^3^ in the cytosol. Since approximately 2 % of the proteome is estimated to be prenylated, PDEδ modulates several cellular processes.^1^, ^2^, ^4^ However, only a small subset of S‐prenylated proteins has been identified as PDEδ cargo so far^5^ such that knowledge about the biological functions of PDEδ is limited. Small‐molecule inhibitors that potently target the PDEδ prenyl‐binding site, for example, deltasonamide 1 (Figure 1), have proven invaluable for the study of PDEδ function.^6^, ^7^, ^8^, ^9^ However, their application is limited by an allosteric interaction of PDEδ with the Arl2/3 GTPases, which results in release of even high‐affinity cargo.^4^, ^8^, ^9^

**Figure 1:**
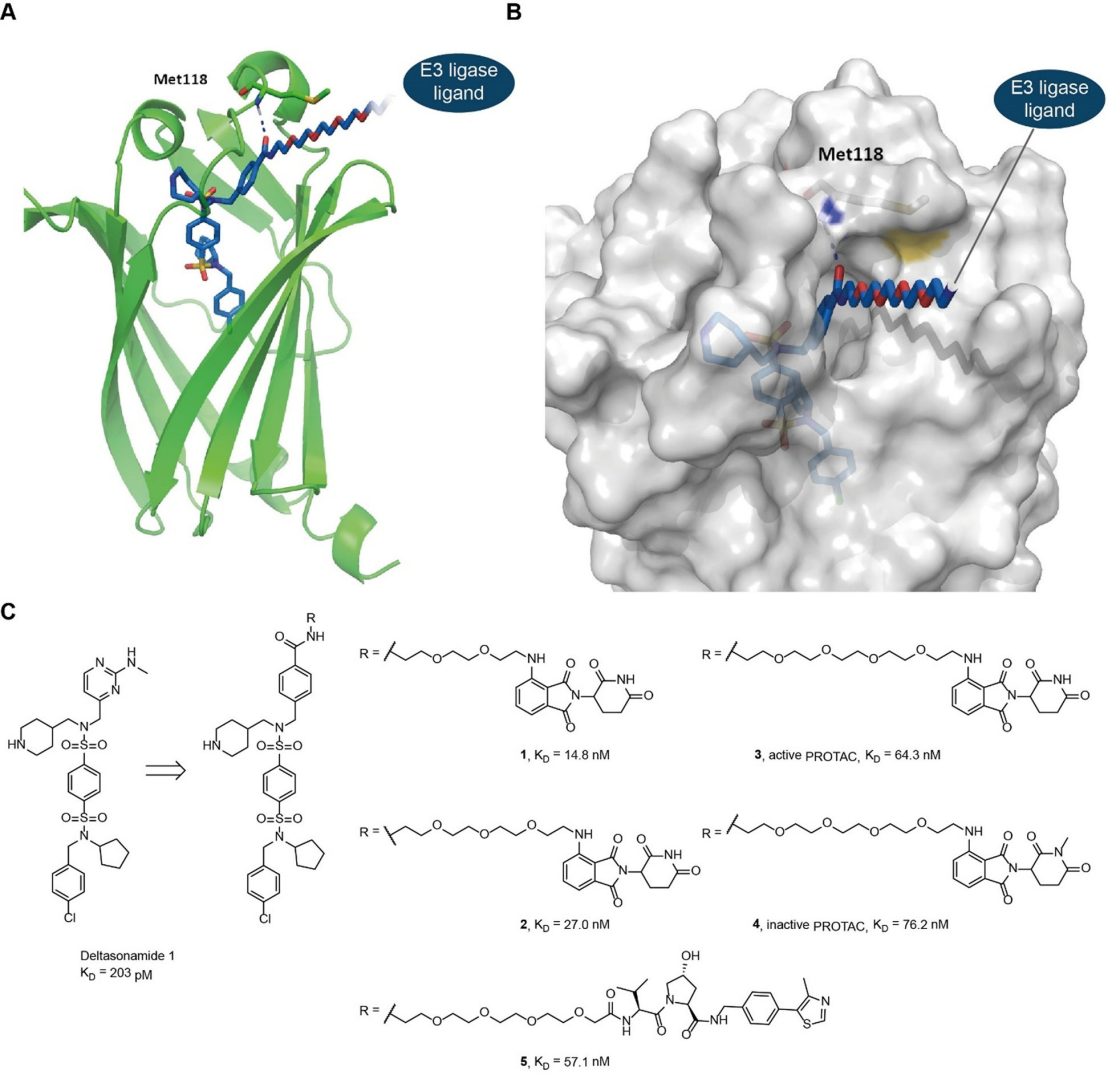
Design of PDEδ PROTAC probes. A, B) Visualization of the PDEδ PROTAC **3** in the binding pocket of PDEδ based on computational modelling (PDB ID: 5ML3). A) The amide C=O forms a hydrogen bond to the backbone NH of Met118. The hydrogen bond is indicated by a dotted line. B) Solvent‐accessible surface of PDEδ around the linker region of PDEδ PROTAC **3**. C) Structure and affinity of the pomalidomide‐based PDEδ PROTACs **1**–**4** and the VHL‐based PROTAC **5**. Affinity for PDEδ was determined by competitive fluorescent polarization analysis.

An alternative approach to inhibition consists of event‐driven pharmacology, for example, small‐molecule‐induced protein degradation. For this purpose, heterobifunctional molecules are employed that bind the protein of interest and recruit an E3 ubiquitin ligase, followed by ubiquitination and subsequent degradation of the targeted protein. Such proteolysis‐targeting chimeras (PROTACs) mediate chemical protein knockdown. Originally introduced by Crews and Deshaies et al.,^10^ this approach has recently gained major attention in both chemical biology and medicinal chemistry research.^11^ While classic inhibitors, like deltasonamide 1, rely on high binding‐site occupancy, PROTACs do not need to bind the target protein permanently. After ternary complex ubiquitination, they can be recycled, that is, they may act catalytically. Therefore, the use of PDEδ PROTACs may be a promising approach to gain new insight into PDEδs biology and function.

Herein, we describe the development of PROTACs that efficiently and selectively reduce PDEδ levels in cells. Surprisingly, application of the PDEδ PROTACs, and by analogy also the PDEδ inhibitor deltasonamide 1, increased the expression of various enzymes involved in lipid metabolism through induction of the sterol response element, resulting in elevated levels of cholesterol precursors. This finding for the first time demonstrates that proper PDEδ function plays a role in the regulation of sterol synthesis.

The picomolar PDEδ inhibitor deltasonamide 1 (Figure 1) binds to the prenyl‐binding pocket of PDEδ, with 10 noncovalent interactions locking the compound into the binding site. The pyrimidine ring is crucial to obtain picomolar affinity. However, the benzyl derivative, which displays 5‐fold lowered affinity, offers a simplified synthesis for the attachment of a linker (Figure 1).^8^ Moreover, PROTAC‐mediated degradation is event‐driven and the low nanomolar affinity of the benzyl analogue for PDEδ would most likely be sufficient to trigger target degradation. Thus, we designed PROTACs based on the benzyl derivative of deltasonamide 1, where the E3 ligase ligand was attached via an oligoethylene glycol linker to the benzoic acid (Figure 1). As the E3 ligand, we chose the immunomodulatory compound pomalidomide, which targets cereblon, a substrate receptor for the cullin 4‐RING E3 ligase complex cullin 4. In addition, we employed a ligand for Von‐Hippel‐Lindau (VHL), the substrate adapter for the E3 ubiquitin ligase cullin 2.^12^ Since PROTAC‐mediated degradation strongly depends on the linker length, which modulates formation of an active ternary complex,^13^ linkers with three different ethylene glycol units were considered to yield PROTAC probes **1**–**3**.

Treatment of Jurkat cells with PROTACs **1**, **2**, and **3** at 1 μm concentration after 24 and 48 h induced degradation of PDEδ with different maximum degradation efficacies (*D*_max_; Figure 2 A and B), with PROTAC **3** being the most efficient (*D*_max_=85 % at 1 μm). In the pancreatic cancer cell line Panc Tu‐I, the PROTACs also downmodulated PDEδ after 24 h treatment. PROTAC **3** caused degradation with a half‐maximal degradation concentration (DC_50_) of 48 nm and a *D*_max_ of 83.4 % (Figure 2 C and D). Degradation of PDEδ appears to depend on E3‐ligase recruitment and subsequent ubiquitination since the inactive PROTAC **4** did not reduce the level of PDEδ (Figure 2 C and D). Additionally, Panc Tu‐I cells showed reduced PDEδ levels after treatment with VHL‐based PDEδ PROTAC **5** (Figure S1 in the Supporting Information). To investigate whether attachment of the linker affects the binding to PDEδ, a competitive fluorescence polarization assay was performed, monitoring displacement of the known PDEδ ligand atorvastatin‐FITC by the PROTACs.^7^ Probes **3** and **4** bound with high affinity to PDEδ (64.3±1.7 nm and 76.2±1.6 nm, respectively) and binding is not affected by CRBN (see Figure 1 C and Figure S2). Furthermore, PDEδ VHL‐PROTAC **5** showed similar high affinity to PDEδ (57.1±1.6 nm; Figure S2).

**Figure 2:**
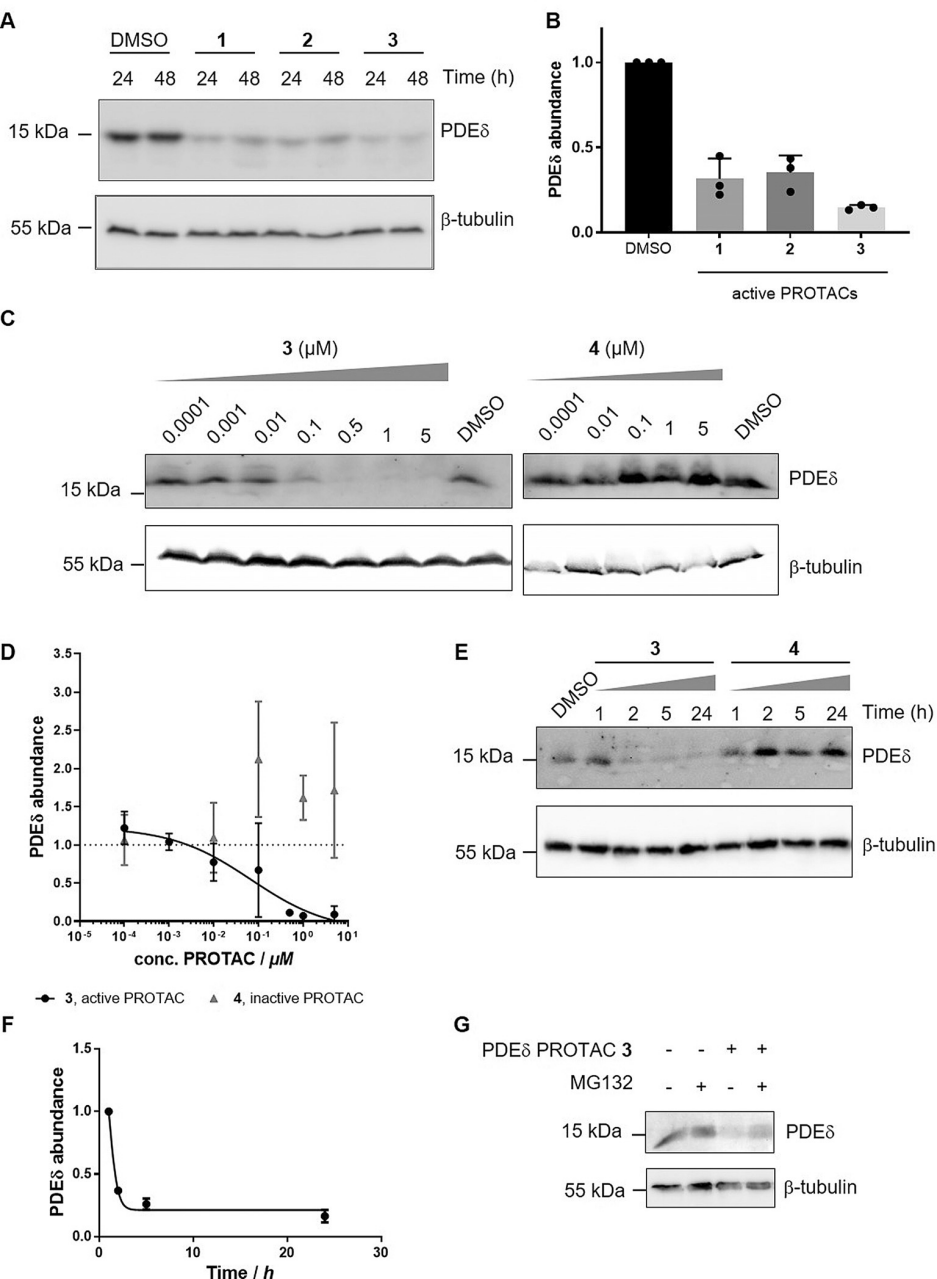
Concentration‐ and time‐dependent degradation of PDEδ by PROTAC **3**. A) Jurkat cells were treated for 24 or 48 h with 1 μm of PROTAC **1**, **2**, or **3** or DMSO as a control. Cells were lysed and proteins were subjected to immunoblotting using specific antibodies for PDEδ and β‐tubulin as a reference protein. B) Quantification of band intensities from (A). Data are mean values±SD (*n*=3). C) Panc Tu‐I cells were treated for 24 h with different concentrations of PROTAC **3** or **4** and DMSO as a control. Cells were lysed and proteins were subjected to immunoblotting using specific antibodies for PDEδ and β‐tubulin as a reference protein. D) Dose–response curve for PROTAC **3** mediated degradation of PDEδ as quantified by immunoblotting. Data are mean values±SD (*n*=3). E) Panc Tu‐I cells were treated for different time periods with 1 μm of the active PROTAC **3** or the inactive PROTAC **4**. Cellular PDEδ levels are visualized using immunoblotting. F) Quantification of PDEδ abundance using immunoblotting after treatment with PROTAC **3**. Data are mean values±SD (*n*=3). G) Panc Tu‐I cells were treated with 1 μm of PROTAC **3** and 10 μm of the proteasome inhibitor MG132 for 3 h. Cell lysate were subjected to immunoblotting to visualize PDEδ and β‐tubulin as a reference protein as described in (C).

After 5 h of treatment with 1 μm of PROTAC **3**, cellular PDEδ levels reached a minimum of 16.4 % (Figure 2 E and F). In contrast, treatment with the inactive PROTAC **4** over 24 h did not reduce the cellular PDEδ content (Figure 2 E and Figure S3).

To demonstrate that PROTAC‐induced PDEδ depletion occurs through proteasomal degradation, cells were treated with PROTAC **3** and the proteasome inhibitor MG132. Blocking the proteasome using MG132 during treatment with PROTAC **3** restored PDEδ levels compared to treatment with PROTAC **3** only (Figure 2 G and S4).^14^ Thus, PROTAC **3** mediated depletion of PDEδ depends on proteasomal activity.

PROTACs should also cause the degradation of any protein that is fused to their target. We generated a HeLa cell line that stably expresses a NanoLuc luciferase‐PDEδ fusion protein. Determination of NanoLuc activity in these cells using the substrate furimazine is a direct readout of NanoLuc, and thus, PDEδ levels. Upon treatment with PROTAC **3**, NanoLuc activity decreased in a concentration‐dependent manner, whereas PROTAC **4** did not affect the activity of NanoLuc (Figure 3 A). Moreover, we monitored the level of PDEδ by means of mCherry‐dependent fluorescence detection in HEK293T cells transiently expressing mCherry‐PDEδ.^2^ This setup allows real‐time analysis of PDEδ levels, that is, PDEδ‐induced degradation by PROTAC **3**. A steady decrease in mCherry fluorescence and thus of PDEδ levels was observed after addition of PROTAC **3**, whereas PROTAC **4** was inactive (Figure 3 B and C).

**Figure 3:**
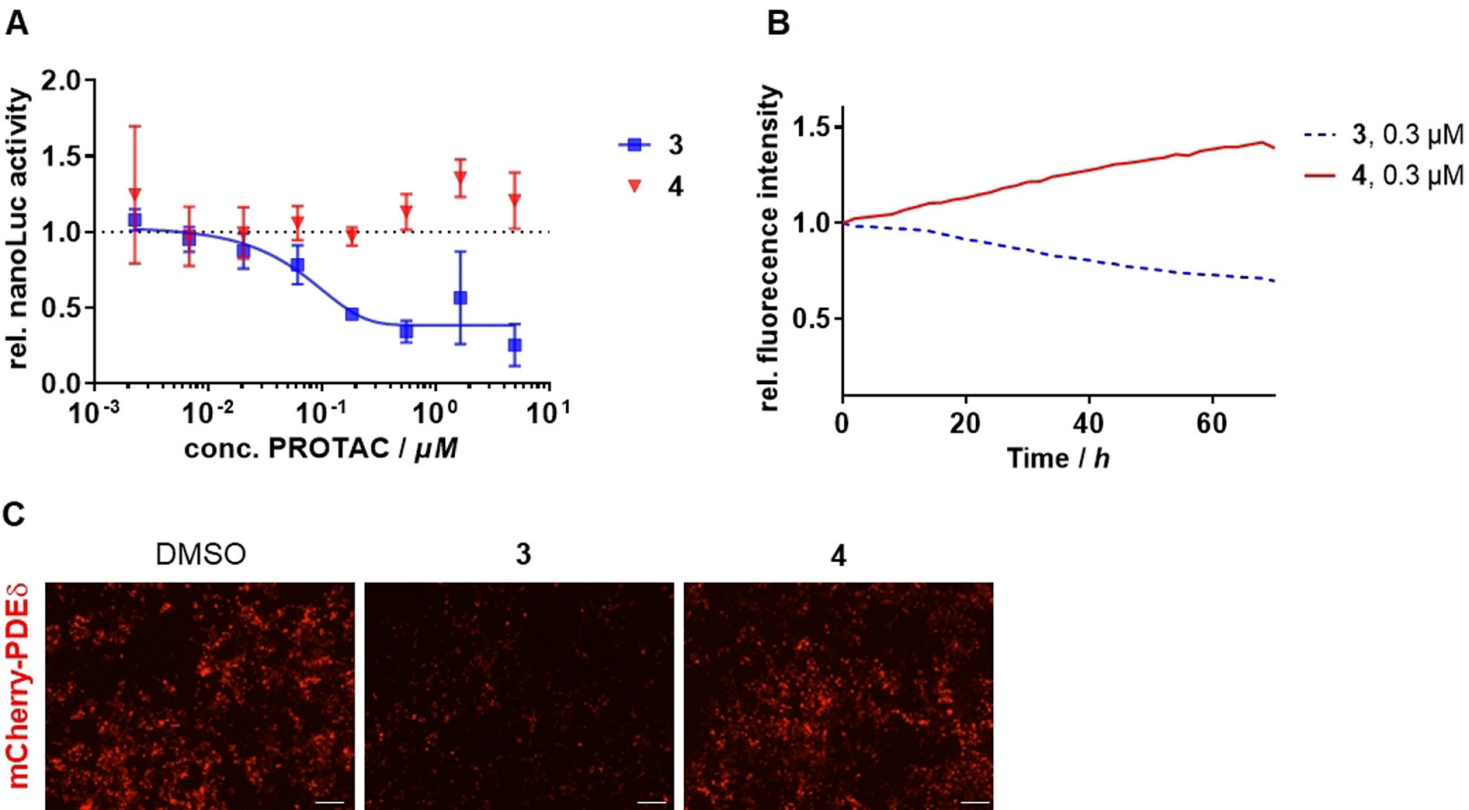
Active PROTAC **3** degrades PDEδ fusion proteins. A) NanoLuc‐expressing HeLa cells were treated with different concentrations of the active PROTAC **3** or inactive PROTAC **4** for 24 h. NanoLuc activity was normalized to the activity of cells that were treated with DMSO. Data are mean values±SD (*n*=3). B) HEK293T cells, which transiently express mCherry‐PDEδ, were treated with the active PROTAC **3** or inactive PROTAC **4**. mCherry intensities were normalized to the DMSO control and related to time 0 (set to 1). C) Representative images from (B) after 24 h of treatment with 1 μm PROTAC **3**, PROTAC **4** or DMSO. Scale bar: 100 μm.

To determine the specificity of PROTACs **3** and **5** for PDEδ depletion, we performed proteome profiling of cells that were treated for 5 h and 24 h with PROTAC **3** and PROTAC **5** in comparison to DMSO‐treated HeLa cells. Furthermore, for comparison, we investigated up‐ and down‐regulation of proteins by the inactive PROTAC **4** and deltasonamide 1 after 24 h. Protein levels were quantified using tandem mass tag (TMT) labeling and mass spectrometry (Figure 4 A).

**Figure 4:**
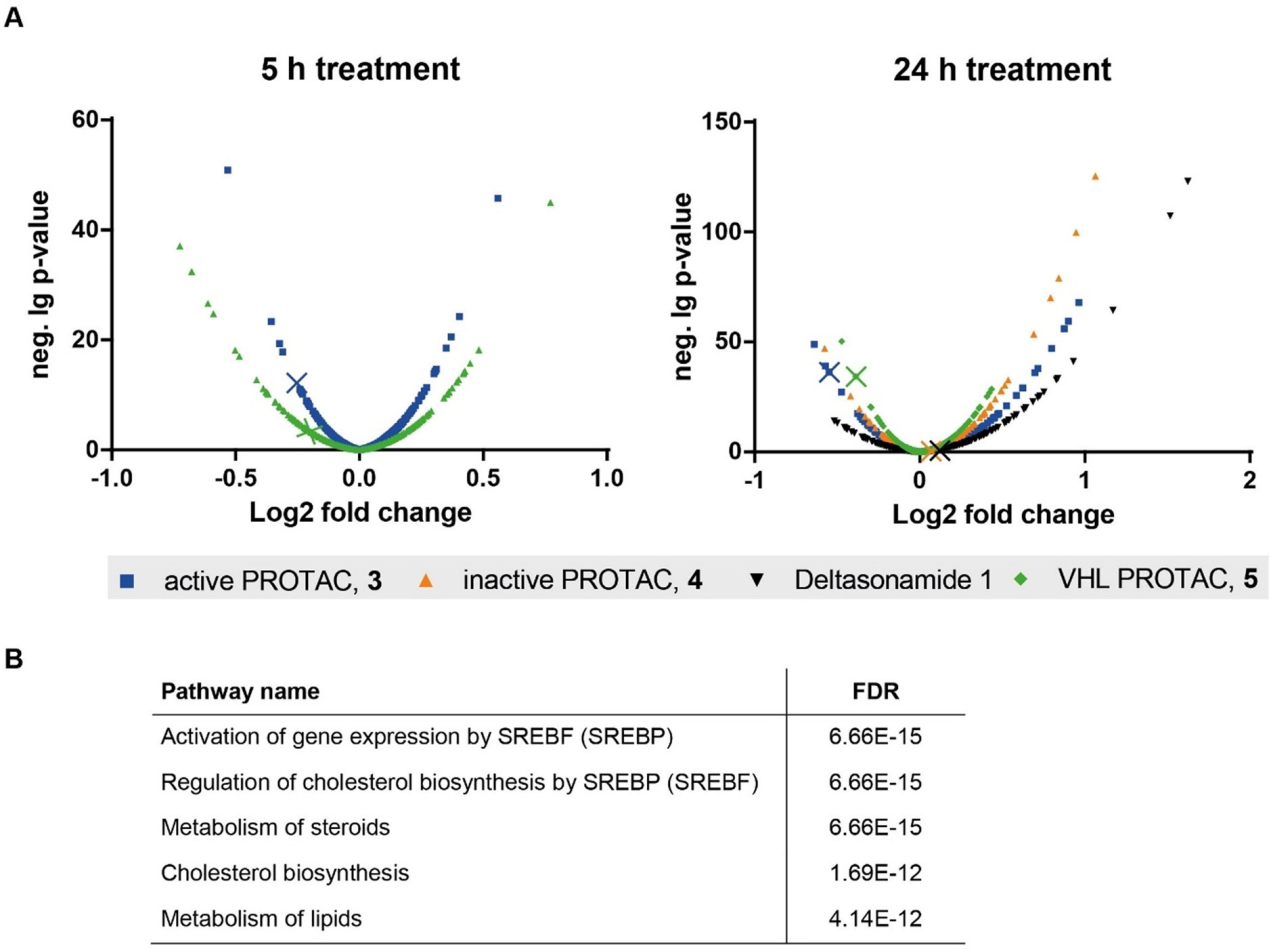
Proteome‐wide tracking of protein degradation by PROTACs **3** and **5**. A) HeLa cells were treated with 1 μm of PROTAC **3**, PROTAC **4**, deltasonamide 1, or VHL‐based PROTAC **5** for 24 h or 1 μm PROTAC **3** or **5** for 5 h. Cellular protein levels were determined using TMT labeling and mass spectrometry. Each dot represents the mean p‐value (n=3). All identified proteins are displayed. Colored crosses point to the abundance of PDEδ in each condition. B) Reactome pathway analysis of the significantly upregulated proteins after treatment with PROTAC **3** for 24 h.

Inspection of all significantly downregulated proteins revealed that PDEδ is the only protein with lower abundance after incubation for 5 h and 24 h with the active PROTAC **3** (Table S1). After 24 h, only the levels of PDEδ and ferritin light chain (FTL) were reduced by the active PROTAC **3** and PROTAC **5** (Figure S5 and Table S2). In contrast, neither the inactive PROTAC **4** nor the parental compound deltasonamide 1 reduced PDEδ levels after 24 h incubation time (Figure S5 and Table S2). These findings demonstrate that the PDEδ‐based PROTACs selectively target PDEδ for degradation.

Interestingly, the abundance of several proteins was higher in PROTAC‐treated cells than in the DMSO‐treated control cells. Analysis of the set of upregulated proteins (Table S3) using the Reactome tool^15^ revealed activation of gene expression by sterol regulatory element binding protein (SREBP), regulation of cholesterol biosynthesis by SREBP, and the metabolism of cholesterol and lipids as the most significantly enriched pathways after 24 h incubation time with the active PROTACs **3** and **5**, the inactive PROTAC **4**, and deltasonamide 1 (Figure 4 B and Table S4). Most of these proteins are enzymes involved in lipid metabolism, in particular enzymes of the mevalonate pathway, for example, HMGCS, ACSS2, MVD, IDl1, which are responsible for cholesterol and isoprenoid precursor synthesis from acetyl‐CoA (Figure S6). These findings indicate that interference with PDEδ function, that is, either chemical inhibition or PROTAC‐mediated degradation, increases the levels of enzymes involved in lipid metabolism.

Most of the identified upregulated proteins are regulated through the sterol regulatory element (SRE), which is bound by sterol regulatory element binding protein (SREBP). At low cellular sterol levels, SREBP binds together with other transcription factors to SRE sites in gene promoters to induce the expression of lipid metabolism enzymes. Upregulation of mevalonate pathway enzymes through SRE by PDEδ‐targeting agents was investigated by means of an SRE‐based reporter gene assay. To this end, HeLa cells were transfected with a firefly luciferase (Fluc) construct under the transcriptional control of SRE. After 24 h of treatment with PROTAC **3** and deltasonamide 1, increased activity of Fluc was observed (Figure 5 A). Deltasonamide 1 displays stronger SRE activation compared to PROTAC **3**, which can be attributed to its very high affinity for PDEδ. The mevalonate pathway product cholesterol inhibits the SRE‐mediated gene expression by binding to SREBP cleavage‐activating protein (SCAP) and sequestering SREBP in the endoplasmic reticulum, thus, suppressing SREBP translocation into the nucleus.^16^ To investigate whether the compounds act upstream or downstream of cholesterol in regulating the SRE response, we simultaneously treated cells with 2.5 μm 25‐hydroxycholesterol, 25 μm cholesterol, and 1 μm of the PDEδ inhibiting agents. In cholesterol‐rich medium, deltasonamide 1 failed to induce SRE‐dependent luciferase expression, which indicates a mode‐of‐action upstream of SREBP regulation by cholesterol (Figure 5 A).

**Figure 5:**
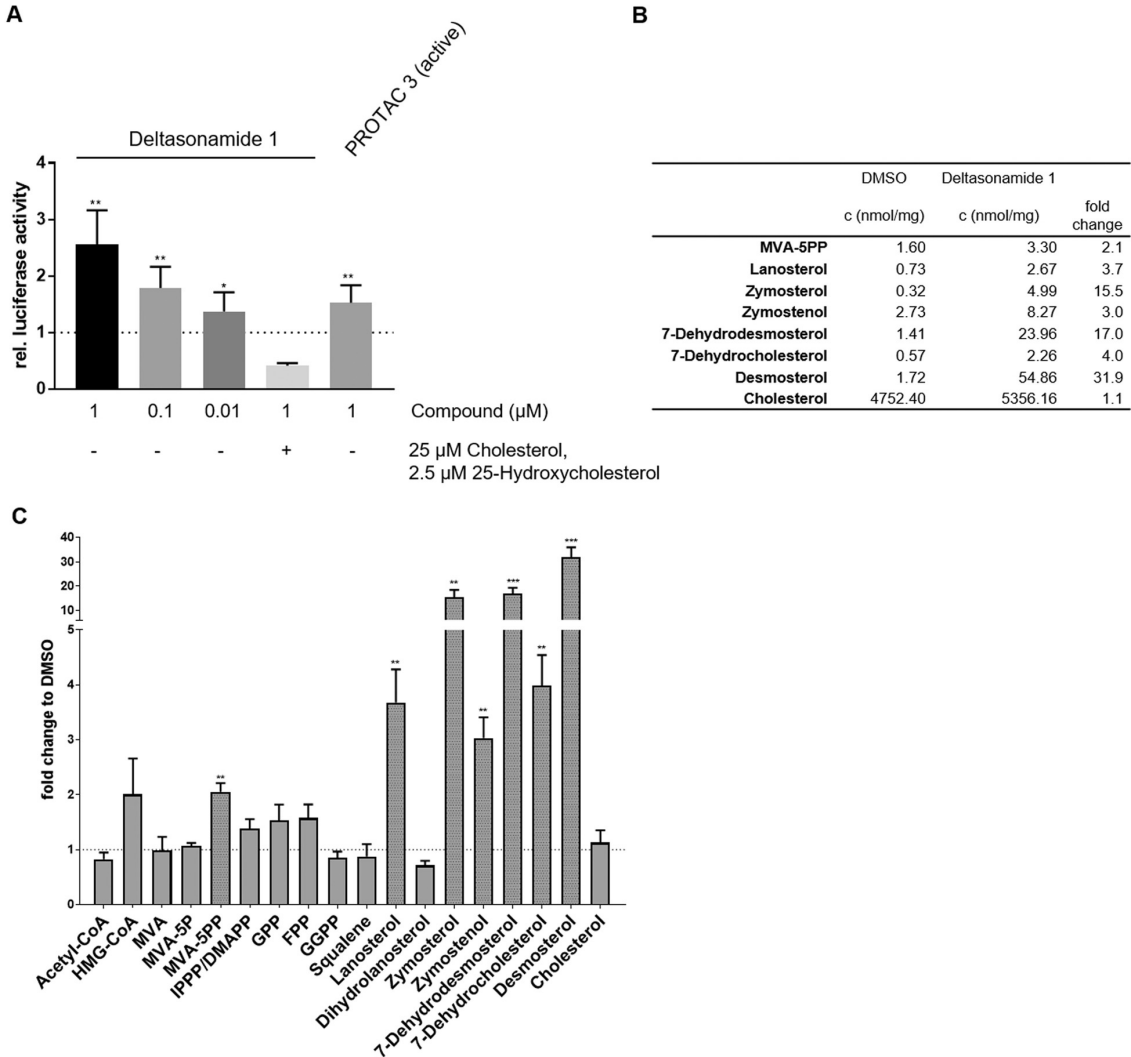
Influence of PDEδ targeting modalities on SRE‐mediated gene expression and the level of lipid metabolites. A) HeLa cells were transfected with a firefly luciferase (Fluc) construct under the control of SRE and a plasmid for constitutive Renilla luciferase (Rluc) expression. Cells were treated with the compounds for 24 h prior to determination of Fluc and Rluc activity. Where indicated, cells were co‐treated with 2.5 μm 25‐hydroxycholesterol and 25 μm cholesterol. Data are mean values±SD (n=3). B) HeLa cells were treated for 24 h with 1 μm deltasonamide 1. Metabolites were quantified using mass spectrometry. Absolut values for significant quantification of metabolites are shown in the table. C) Obtained values were normalized to the values of DMSO‐treated cells. Data are mean values±SD (*n*=3). * *p* ≤0.05, ** *p* ≤0.01, *** *p* ≤0.001, MVA=mevalonic acid, IPPP=isopropyl pyrophosphate, HMG‐CoA=3‐hydroxy‐3‐methylglutaryl‐CoA, DMAP=dimethylallyl pyrophosphate, GPP=geranyl pyrophosphate, FPP=farnesyl pyrophosphate.

Since increased levels of mevalonate‐pathway enzymes should lead to an increase in lipid‐metabolism‐related metabolites, HeLa cells were treated for 24 h with 1 μm deltasonamide 1, and metabolite levels were subsequently quantified by means of mass spectrometry. This metabolic analysis revealed that the compound induces elevation of mevalonic acid‐5‐pyrophosphate (MVA‐5PP, 2.1‐fold), lanosterol (4‐fold), zymosterol (16‐fold), zymostenol (3‐fold), 7‐dehydrodesmosterol (17‐fold), 7‐dehydrocholesterol (4‐fold), and desmosterol (32‐fold) levels (Figure 5 B and C). Thus, inhibition of PDEδ with deltasonamide 1 leads to the accumulation of cholesterol precursors. In untreated cells, these precursors are present at low concentrations. We did not detect a significant difference in the level of cholesterol, however, the cellular concentration of cholesterol is up to 4000‐fold higher compared to its precursors and may not be subject of further increase (Figure 5 B).

In conclusion, we have developed new PROTACs for the efficient chemical depletion of PDEδ. These PROTACs hold promise as viable tools for further analysis of the biological functions of PDEδ.

## Conflict of interest

The authors declare no conflict of interest.

## Supporting information

As a service to our authors and readers, this journal provides supporting information supplied by the authors. Such materials are peer reviewed and may be re‐organized for online delivery, but are not copy‐edited or typeset. Technical support issues arising from supporting information (other than missing files) should be addressed to the authors.

Supplementary
