# Supplementary material for: Development of a PDEδ‐Targeting PROTACs that Impair Lipid Metabolism
Source: Angew Chem Int Ed Engl. 2020 Jan 22;59(14):5595–601. doi: 10.1002/anie.201913904 (PMC7154537; doi:10.1002/anie.201913904)

Angewandte Chemie
Eine Zeitschrift der Gesellschaft Deutscher Chemiker

Supporting Information

Development of a PDEδ-Targeting PROTACs that Impair Lipid Metabolism

*Michael Winzker, Alexandra Friese, Uwe Koch, Petra Janning, Slava Ziegler, and Herbert Waldmann**

anie_201913904_sm_miscellaneous_information.pdf

Supporting Information

Supporting Figures

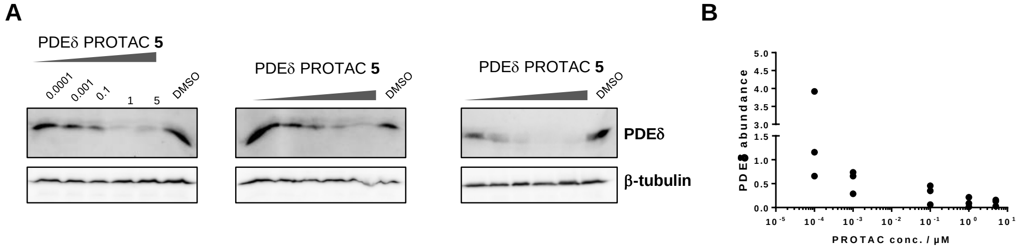

Figure S1: Cellular depletion of PDEδ by a VHL-based PROTAC 5. (A) Detection of PDEδ levels by means of immunoblotting using Panc Tu-I cells treated with different concentrations of VHL PROTAC **5** for 24 h. β-tubulin levels were detected as loading control. (B) Band intensities from B (n=3) were normalized to the levels of β-tubulin and values were then related to the DMSO sample (which was set to 1). Results of all three independent experiments are shown.

A

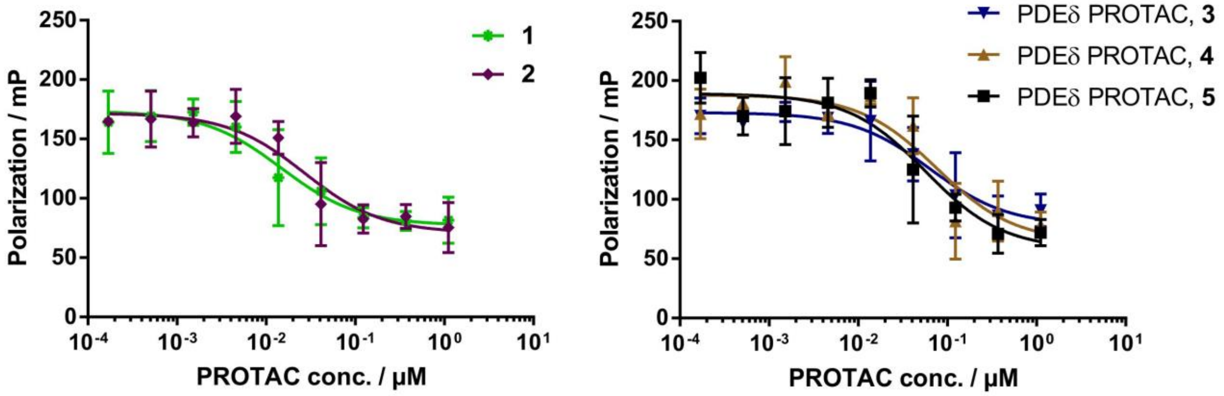

B

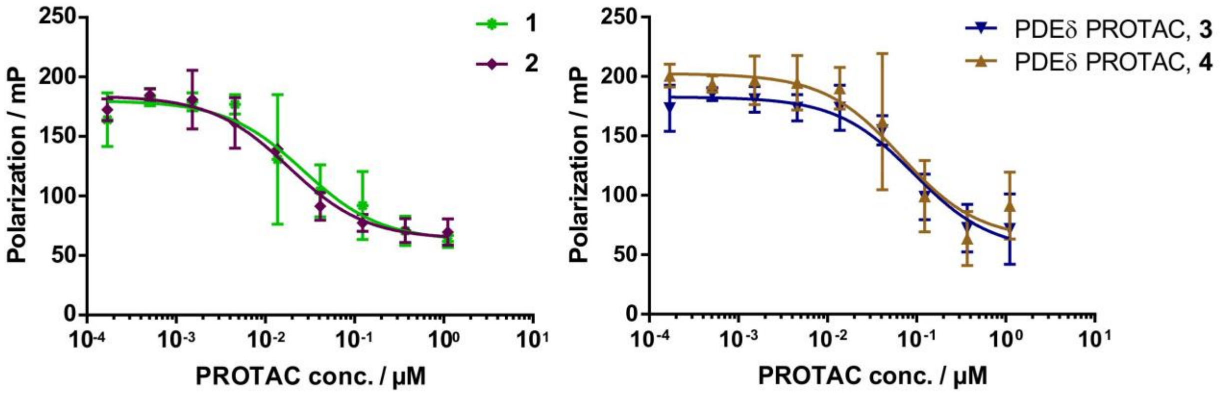

C

	**1**	**2**	PROTAC, **3**	PROTAC, **4**	PROTAC, **5**
$\mathbf{K_{D,PDE\delta}}$	14.8 ± 1.7 nM	27.0 ± 1.5 nM	64.3 ± 1.7 nM	76.2 ± 1.6 nM	57.1 ± 1.6 nM
**+ CRBN**	26.6 ± 1.6 nM	18.4 ± 1.3 nM	92.9 ± 1.4 nM	67.0 ± 1.6 nM	-

Figure S2: Displacement assay to determine apparent K_D values of the compounds. (A) PDEδ was incubated with FITC-labelled atorvastatin, a PDEδ-binder with nanomolar affinity. PROTAC probes were titrated to compete atorvastatin-FITC out of PDEδs pocket and determine the apparent K_D values. (B) Apparent K_D determination in presence of CRBN. (D) Mean apparent K_D values (n=3) of PDEδ PROTAC (**1**-**5**).

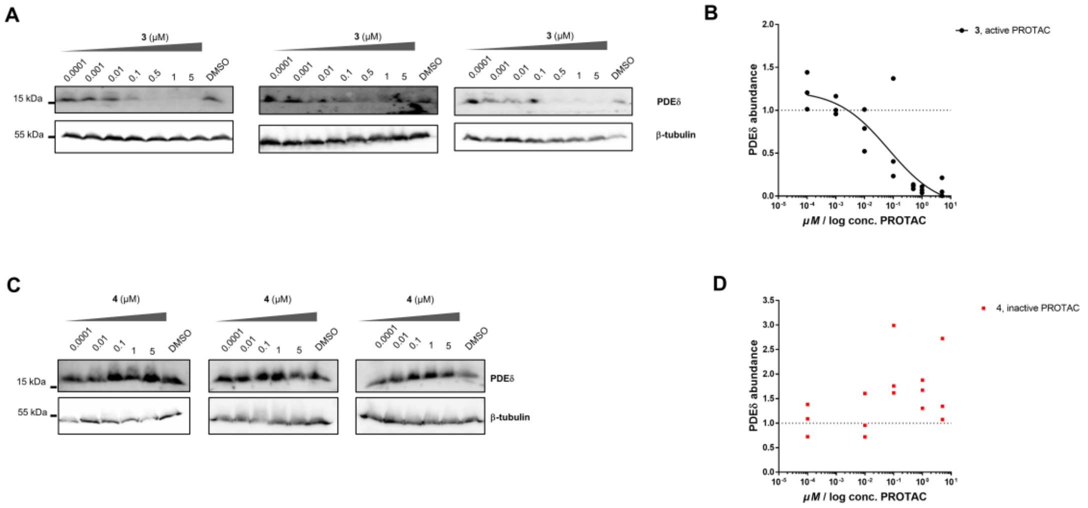

Figure S3: PDEδ PROTAC-mediated depletion of cellular PDEδ. (A) Panc Tu-I cells were treated for 24 h with different concentrations of PROTAC **3** and DMSO as a control. Cells were lysed and proteins were subjected to SDS-PAGE and immunoblotting using specific antibodies for PDEδ and β-tubulin as a reference protein. Results of all three independent experiments are shown. (B) Dose-response curve for PROTAC **3**-mediated degradation of PDEδ. Band intensities were quantified, normalized to the intensity of the respective band for β-tubulin and then related to the DMSO sample of the respective treatment time, which was set to 1. Each dot represents a biological replicate. (C) Panc Tu-I cells were treated for 24 h with different concentrations of PROTAC **4** and DMSO as a control. Cells were lysed and proteins were subjected to SDS-PAGE and immunoblotting using specific antibodies for PDEδ and β-tubulin as a reference protein. Results of all three independent experiments are shown. (D) Dose-response curve for PROTAC **4**-mediated degradation of PDEδ. Band intensities were quantified, normalized to the intensity of the respective band for β-tubulin and then related to the DMSO sample of the respective treatment time, which was set to 1. Each dot represents a biological replicate.

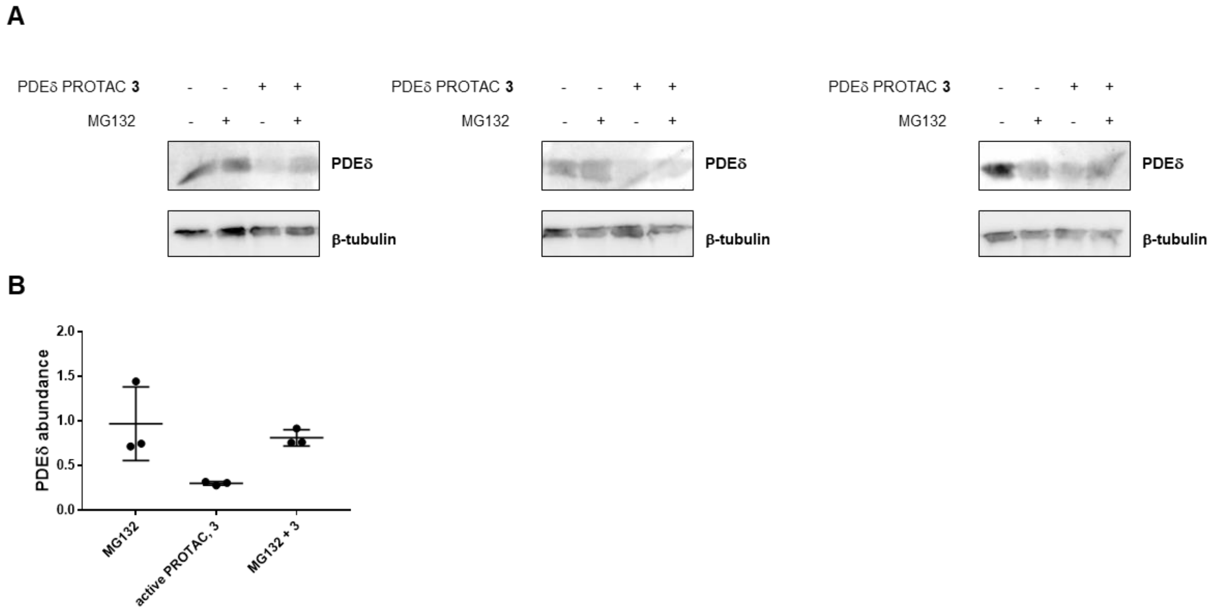

Figure S4: Proteasome inhibitor blocks PDEδ PROTAC-mediated degradation. (A) Panc Tu-I cells were treated with 1 μM of PDEδ PROTAC **3** in presence of MG132 a proteasome inhibitor. Then, cell lysate was subjected to SDS-PAGE followed by immunoblotting to detect cellular levels of PDEδ and a reference. (B) Band intensities were normalized to the reference and the intensity for DMSO-treated cells was set to 1.

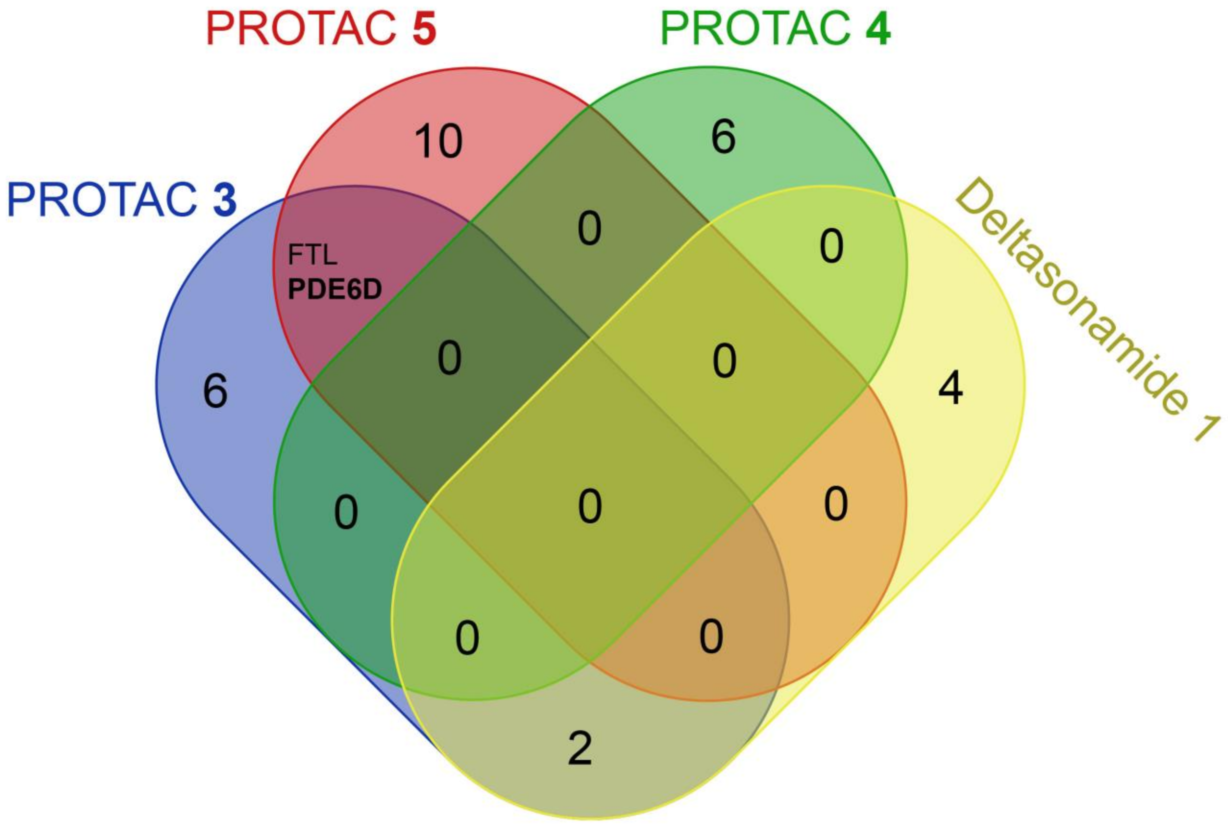

Figure S5: Specificity of PDEδ degradation by PROTAC 3 and PROTAC 5. Venn diagram representing the intersection of downregulated proteins by PROTAC3, PROTAC4, PROTAC 5 and Deltasonamide 1 after an incubation for 24 h. The Venn diagram was generated using http://bioinformatics.psb.ugent.be/webtools/Venn/.

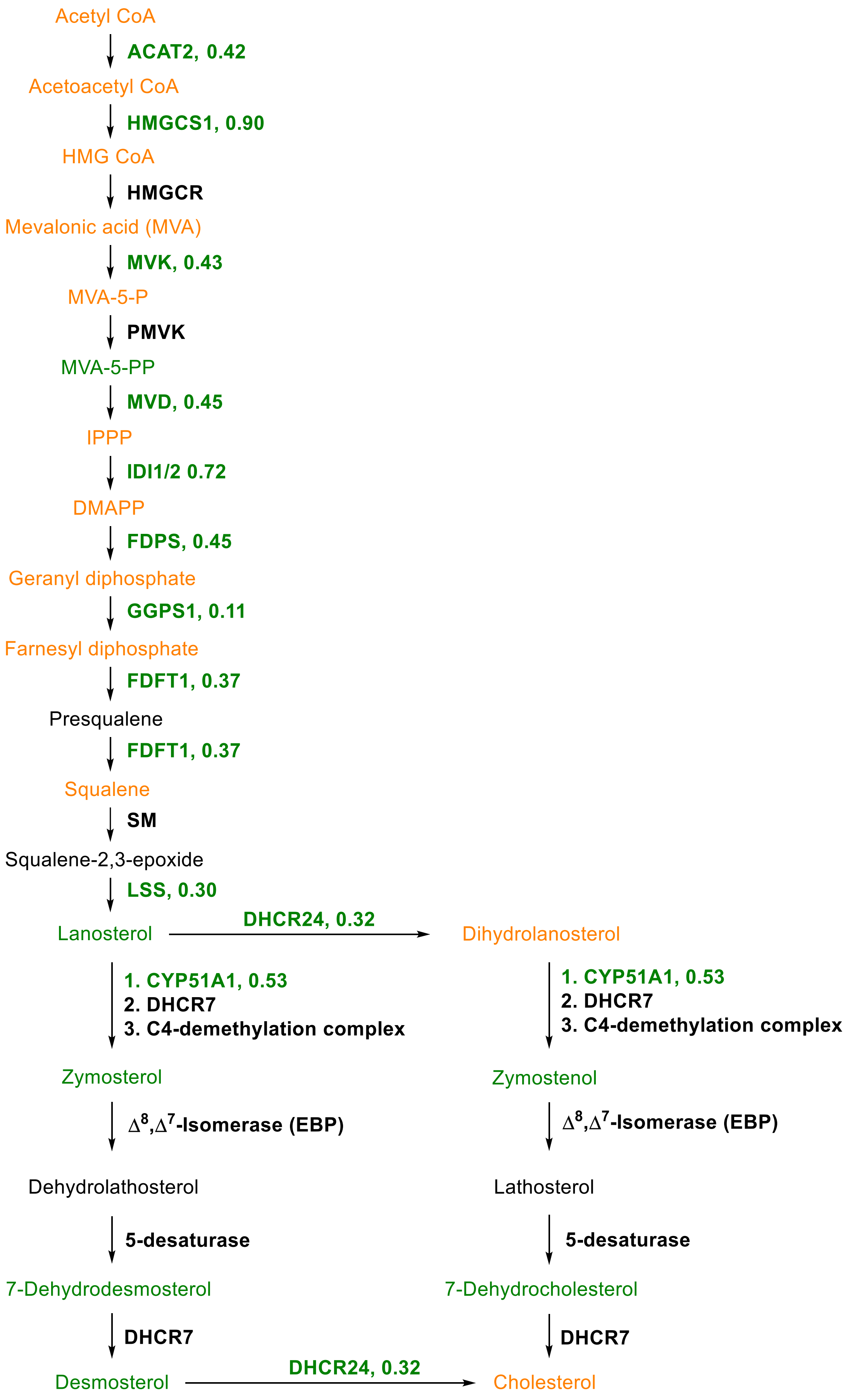
Acetyl CoA
ACAT2, 0.42
Acetoacetyl CoA
HMGCS1, 0.90
HMG CoA
HMGCR
Mevalonic acid (MVA)
MVK, 0.43
MVA-5-P
PMVK
MVA-5-PP
MVD, 0.45
IPPP
IDI1/2 0.72
DMAPP
FDPS, 0.45
Geranyl diphosphate
GGPS1, 0.11
Farnesyl diphosphate
FDFT1, 0.37
Presqualene
FDFT1, 0.37
Squalene
SM
Squalene-2,3-epoxide
LSS, 0.30
Lanosterol
DHCR24, 0.32
Dihydrolanosterol
1. CYP51A1, 0.53
2. DHCR7
3. C4-demethylation complex
1. CYP51A1, 0.53
2. DHCR7
3. C4-demethylation complex
Zymosterol
Zymostenol
Δ^8,Δ^7-Isomerase (EBP)
Δ^8,Δ^7-Isomerase (EBP)
Dehydrolathosterol
Lathosterol
5-desaturase
5-desaturase
7-Dehydrodesmosterol
7-Dehydrocholesterol
DHCR7
DHCR7
Desmosterol
DHCR24, 0.32
Cholesterol

Figure S6: Upregulation of enzymes and metabolites in the mevalonate and cholesterol biosynthesis pathway. HeLa cells were treated with 1 µM of compound for 24 h and were subjected to either proteome profiling or metabolomics. Protein levels were quantified for cells treated with active PROTAC **3** and metabolites were quantified of cells treated with Deltasonamide 1. Green: upregulation compared to DMSO-treated cells; orange: no significant change compared to DMSO-treated cells.; black: not identified; number: fold change to DMSO-treated cells.

Supporting Tables

Table S1: Significantly downregulated proteins. HeLa cells were treated for 5 h or 24 h with 1 µM of PROTAC **3** and were subjected to a proteome-wide analysis. Significant boarders were set to $p < 0.00001$ (Benjamini-Hochberg corrected).

Names	Proteins
**PROTAC 3 5 h & PROTAC 3 24 h**	**PDE6D**
**PROTAC 3 5 h**	AKAP1<br>ANKIB1<br>APPL2<br>C11orf84<br>CASP9<br>CRTC1<br>ENO1<br>INTS2<br>ITSN2<br>KIF7<br>KLHL5<br>LDOC1L<br>MZT2B<br>PLEKHG3<br>WDR33<br>ZNF592
**PROTAC 3 24 h**	AMD1<br>CDK11A<br>CEP72<br>DPH7<br>ERBB2IP<br>FAM160A1<br>FLNA<br>FTL

Names	Proteins
	GPN2<br>HES1<br>HPS3<br>MARS2<br>MED15<br>NCSTN<br>PRKAB1<br>PTCD1<br>RCHY1<br>RILPL2<br>SHPRH<br>TOP3A<br>TPM2<br>ZFP91<br>ZNF384

Table S2: Significantly downregulated proteins. HeLa cells were treated for 24 h with 1 µM of PROTAC **3, 4, 5** and Deltasonamide 1 and were subjected to a proteome-wide analysis. Significant boarders were set to $p < 0.00001$ (Benjamini-Hochberg corrected).

**Names**	**Proteins**
**PROTAC 3 & PROTAC 5**	FTL<br>**PDE6D**
**Deltasonamide 1 & PROTAC 3**	FLNA<br>TPM2
**PROTAC 3**	CDK11A<br>CEP72<br>FAM160A1<br>HES1<br>RILPL2<br>ZFP91
**PROTAC 5**	ARMC1<br>AVL9<br>BAHCC1<br>BRF1<br>DAG1<br>FEM1A<br>GNAS<br>NFAT5<br>PROCR<br>TAF8
**PROTAC 4**	COL4A2<br>FASTKD3<br>hCG_2043597<br>LZTR1
**Deltasonamide 1**	ARL6IP4<br>NOLC1<br>RPL11<br>TPM1

Table S3. Significantly upregulated proteins. HeLa cells were treated for 24 h with 1 µM of PROTAC 3, 4, 5 and Deltasonamide 1 and were subjected to a proteome-wide analysis. Significant boarders were set to $p < 0.00001$ (Benjamini-Hochberg corrected).

PROTAC3	PROTAC4	Deltasonamide 1	PROTAC 5
ACAT2	ACAT2	ACAT2	ACSS2
ACSS2	ACSS2	ACLY	APP
AP3B2	APOB	ACSL4	CKMT1A
APP	APP	ACSS2	COL1A1
ARHGEF12	CALCOCO2	APP	CYP51A1
ARMC7	CDC25C	BANF1	DTNBP1
BANF1	CETN2	CETN2	FBXW9
CYP51A1	CYR61	CYP51A1	FDFT1
DHCR24	DDRGK1	DHCR24	HMGCS1
FABP3	FASN	FASN	IDI1
FADS2	FDFT1	FDFT1	LRRC45
FASN	FDPS	FDPS	MVK
FDFT1	GLG1	FRA10AC1	NUB1
FDPS	GOLIM4	GABARAPL2	RAB27A
GEMIN6	GPNMB	HMGCS1	RINT1
GPRASP2	GRN	IDH1	SDF2
HMGCS1	HMGCS1	IDI1	TUBGCP6
IDI1	HMOX1	IFIT1	
JMY	IDH1	LSS	
LSS	IDI1	MMAB	
MVD	LPIN1	MVD	
MVK	LSS	MVK	
MYO6	MMAB	NEU1	
NEU1	MVD	NSDHL	
PCSK9	MVK	PCSK9	
PCYT2	NEU1	PCYT2	
PDE2A	PCSK9	PDE2A	
PRAF2	PCYT2	PRUNE2	
RSAD1	PNMA2	RRAGC	
STXBP4	PRUNE2	STXBP1	
TEFM	QPRT	TPRG1L	
TPRG1L	RHOB	WBP2	
WBP2	STXBP1	WDR63	
ZSWIM8	TRAF6		
	WBP2		

Table S4: Reactome pathway analysis. HeLa cells were treated for 24 h with 1 µM PROTAC 4, PROTAC 5 and Deltasonamide 1 and were subjected to a proteome-wide analysis. Significantly ($p < 0.00001$) upregulated proteins were analyses by Reactome pathway analysis.[1] False discovery rates are listed for the top five pathways.

**Pathway name**	**PROTAC 4**	**PROTAC 5**	**Deltasonamide 1**
Activation of gene expression by SREBF (SREBP)	1.71E-14	1.63E-14	7.99E-15
Regulation of cholesterol biosynthesis by SREBP (SREBF)	1.71E-14	2.43E-14	7.99E-15
Metabolism of steroids	5.72E-14	6.49E-9	7.99E-15
Cholesterol biosynthesis	1.46E-8	7.14E-6	5.4E-14
Metabolism of lipids	2.91E-7	4.42E-4	1.78E-12

Experimental Section

Fluorescent polarization-based PDEδ displacement assay

Binding affinity to PDEδ was verified and quantified by using a displacement assay as described previously.[2] Briefly, FITC-labelled Atorvastatin (Lipitor®) a nanomolar PDEδ binder was employed. Therefore, 10 nM labelled Atorvastatin was incubated with 15 nM His_6-PDEδ and different concentrations of compound. The influence of CRBN binding to the PROTAC was determined in presence of 15 nM CRBN. The changes in fluorescence polarization were plotted against the inhibitor concentration and fitted with GraphPad Prism 7 using three parameter non-liner regression fit to determine the apparent K_D values.

Cell culture

HeLa and Panc Tu-I cells were grown in Dulbecco's Modified Eagle's Medium (4.5 g/L glucose, 4 mM glutamine) supplemented with 10% fetal bovine serum, 10 mM sodium pyruvate and non-essential amino acids (PAN-Biotech). Jurkat cells were grown in RPMI 1640 medium supplemented with 10% fetal bovine serum and non-essential amino acids (PAN-Biotech). All cell lines were cultivated in a humidified incubator at 37 °C and 5% CO_2. Adherent cells were splitted every 2-3 days to never reach confluency higher than 80%. Jurkat cells were maintained in a cell concentration between 1 x 10^5 and 1 x 10^6 viable cells/mL.

Cell lysate preparation

For immunoblotting, cells were washed twice with ice-cold PBS and lysed using 2x Laemmli buffer without reducing agent and bromphenol blue. Obtained lysates were sonicated and protein concentration was determined using DC™ Protein Assay (Bio-Rad). Prior to SDS-PAGE 5% (v/v) DTT and 0.05% (w/v) bromphenol blue was added to each lysate.

For proteome profiling, cells were washed once with ice-cold PBS and trypsinized for 5 min. Detached cells were washed twice with ice-cold PBS and centrifuged at 500xg for 5 min. The resulting cell suspension was dissolved in 500 µL PBS containing protease inhibitors and

subjected to five freeze-thaw cycles. After 15 min at 15000xg at 4 °C, protein concentration was determined using the DC™ assay (Bio-Rad).

Immunoblotting

Cell lysate corresponding to 200 µg protein was subjected to SDS polyacrylamide gel electrophoresis (SDS-PAGE). Proteins were then transferred onto a PVDF membrane using tank blotting for 1 h at 100 V with ice-cold transfer buffer (14.4% (w/v) glycine, 3.03% (w/v) Tris, 10% methanol). Membranes were blocked with Odyssey® Blocking Buffer (diluted 1:2 with PBS) for 30 min. Membranes were incubated overnight at 4 °C using the primary antibodies in Odyssey® Blocking Buffer (diluted 1:2 with PBS and supplemented with 0.1% (v/v) Tween-20). The following antibodies were used: anti-PDEδ 1:200 (Thermo Fisher Scientific, PA5-22008), anti-β-tubulin 1:2000 (abcam, ab6046), anti-HMGCS 1:100 (abcam, ab87246), anti-β-actin 1:2000 (abcam, ab8229). After washing thrice with PBS-T membranes were incubated with secondary antibodies that were coupled to IR dyes (LI-COR). Band were detected using ChemDocMP Imaging System (Bio-Rad). Quantification of the fluorescence intensity of each band was performed using ImageLab (Bio-Rad). Band intensities were quantified, normalized to the intensity of the respective band for β-tubulin and related to the DMSO sample value (which was set to 1) for the respective time point.

SRE Reporter gene assay

Prior to transfection, 10,000 HeLa cells were seeded per well in a 96-well plate and incubated overnight. The next day cells were transfected using Lipofectamine 2000 according to manufacturer's protocol. Cells were transfected with the reporter plasmid pSynSRE-T-Luc (gift from Timothy Osborne; Addgene plasmid #60444 ; http://n2t.net/addgene:60444 ; RRID:Addgene_60444)[3] and a *Renilla* luciferase construct under the control of TK promoter. 24h post transfection cells were treated with the respective compound. Luciferase activities were measured using the Dual-Luciferase® Reporter Assay System (Promega) according to

manufacturer's protocol. Briefly, medium was exchanged with 50 µL 1x passive lyses buffer and samples were incubated for 30 min. To 10 µL of the cell lysate and 10 µL of firefly luciferase substrate were mixed and the luminescence was detected. Afterwards, *Renilla* luciferase activity was determined by adding 10 µL substrate Coelenterazine and firefly luciferase inhibitor. Values for Fluc were normalized to the respective Rluc values and were related to the value of cells that were treated with DMSO (set to 1).

Live cell imaging of mCherry-PDEδ

Prior to treatment 2,000 HeLa cells were seeded into 96-well plates. Cells were transfected with an mCherry-PDEδ expressing plasmid (gift from Philippe I.H. Bastiaens)[4] using Lipofectamine 2000 according to manufacturer´s protocol. After one day, cells were treated with the compound and fluorescence intensity was detected using the IncuCyte® S3 Live Cell Analysis System.

Establishment of HeLa stably expressing NanoLuc®-PDEδ fusion protein

PDEδ was cloned into the pFN31K vector by using the restriction sites AsiSI and PmeI. Then, cells were transfected with Fugene HD (Promega E4881) using manufacturers protocols. Cells were grown in medium containing 500-800 µg/ml of G418 to induce construct insertion. The selected clone showed constant expression of the fusion protein analysed by wester blotting.

Detection of NanoLuc®-PDEδ fusion protein

One day prior to treatment, 2,000 HeLa cells stably expressing nanoLuc-PDEδ were seeded per well into 96-well plates. After 24 h the medium was exchanged with 100 µL DMEM containing the respective compound. NanoLuc activity was measured after 24 h of compound treatment using the Nano-Glo® Luciferase Assay System (Promega).

Quantification of metabolites

The quantification of selected lipid metabolites was carried out by The Metabolomics Innovation Centre in Canada (TMIC). One day prior to treatment, 400,000 cells were seeded into a 10 mm dish. Cells were treated for 24 h with 1 µM Deltasonamide 1, washed once with ice-cold PBS and

trypsinized. Afterwards cells were centrifuged for 5 min at 300xg and separated from the supernatant. After two washing steps with PBS, supernatants were removed, cells snap frozen in liquid nitrogen and shipped to TMIC on dry ice.

Sample preparation for mass spectrometry

One day prior to treatment 400,000 HeLa cells were seeded into a 10-mm dish. Then, the medium was exchanged with DMEM containing 1 µM of compound. After 24 h of incubation, the medium was removed, and cells were washed once with PBS. Cells were detached and cell suspensions were washed twice with ice-cold PBS followed by centrifugation. Cells were resuspended in 300 µL PBS containing protease inhibitors. After seven freeze-thaw cycles cells were centrifuged for 15 min and 15,000xg. Supernatants were collected and protein concentration was determined using a Bradford assay (Bio-Rad).

75 µL of 2 g/L cell lysate were mixed with 75 µL 200 mM Triethylammonium bicarbonate buffer (TEAB)-Buffer. After addition of 7.5 µL TCEP and incubation at 55 °C for 30 min samples were treated with 7.5 µL iodoacetamide (375 mM) for 30 min in the dark. Proteins were precipitated by adding 900 µL prechilled acetone and incubation at -20 °C overnight. The next day samples were centrifuged for 10 min at 8,000xg and 4 °C and supernatants were removed. The dry protein pellet was dissolved in TEAB buffer containing trypsin, which was used according to manufacturer's protocol. After incubation overnight at 37 °C samples were labeled with TMT label (TMT10plex, # 90110 or TMT6plex, #90066 ThermoFisher Scientific) according to the manufacturer's instruction. All experiments were performed in biological triplicates.

Mass spectrometry (MS)

Prior to nanoHPLC-MS/MS analysis samples were fractionated into 10 fractions on a C18 column using high pH conditions to reduce the complexity of the samples and thereby increasing the number of quantified proteins. Therefore, samples were dissolved in 120 µl of 20 mM ammonium formate (NH_4COOH) at pH 11, followed by incubation in an ultra-sonicator for 2 min, subsequent vortexing for 1 min and centrifugation at 8,000xg for 3 min at room temperature. 50 µl of the

supernatant were injected onto a XBridge C18 column (130 Å, 3.5 µm, 1mm x 150 mm) using a U3000 capHPLCSystem (ThermoFisher scientific, Germany). Separation was performed at a flow rate of 50 µl/min using 20 mM NH4COO pH 11 in water as solvent A and 40% 20 mM NH4COO pH 11 in water premixed with 60% acetonitrile as solvent B. Separation conditions were 95% solvent A / 5% solvent B isocratic for the first 10 min, to desalt the samples, followed by a linear gradient up to 25% in 5 min, a second linear gradient up to 65% solvent B in 60 min, and a third linear gradient up to 100% B in 10 min. Afterwards the column was washed at 100% solvent B for 14 min and re-equilibrated to starting conditions. Detection was carried out at a valve length of 214 nm. The eluate between 15 and 100 min was fractionated into 10 fractions (30 s per fraction, circular fractionation using 10 vials). Each fraction was dried in a SpeedVac at 30°C until complete dryness and subsequently subjected to nanoHPLC-MS/MS analysis.

For nanoHPLC-MS/MS analysis samples were dissolved in 20 µl of 0.1% TFA in water and 3 µl were injected onto an UltiMateTM 3000 RSLCnano system (ThermoFisher scientific, Germany) online coupled to a Q Exactive™ HF Hybrid Quadrupole-Orbitrap Mass Spectrometer equipped with a nanospray source (Nanospray Flex Ion Source, Thermo Scientific). All solvents were LC-MS grade. To desalting the samples, they were injected onto a pre-column cartridge (5 µm, 100 Å, 300 µm ID * 5 mm, Dionex, Germany) using 0.1% TFA in water as eluent with a flow rate of 30 µL/min. Desalting was performed for 5 min with eluent flow to waste followed by back-flushing of the sample during the whole analysis from the pre-column to the PepMap100 RSLC C18 nano-HPLC column (2 µm, 100 Å, 75 µm ID × 50 cm, nanoViper, Dionex, Germany) using a linear gradient starting with 95% solvent A (water containing 0.1 % formic acid) / 5% solvent B (acetonitrile containing 0.1% formic acid) and increasing to 60% solvent A 0.1% formic acid / 40% solvent B in 120 min using a flow rate of 300 nL / min. Afterwards the column was washed (95% solvent B as highest acetonitrile concentration) and re-equilibrated to starting conditions.

The nano-HPLC was online coupled to the Quadrupole-Orbitrap Mass Spectrometer using a standard coated SilicaTip (ID 20 µm, Tip-ID 10 µM, New Objective, Woburn, MA, USA). Mass

range of m/z 300 to 1650 was acquired with a resolution of 60000 for full scan, followed by up to 15 high energy collision dissociation (HCD) MS / MS scans of the most intense at least doubly charged ions using a resolution of 30000 and a NCE energy of 35 %.

Data evaluation was performed using MaxQuant software (v.1.6.1.0)[5] including the Andromeda search algorithm and searching the human reference proteome of the Uniprot database. The search was performed for full enzymatic trypsin cleavages allowing two miscleavages. For protein modifications carbamidomethylation was chosen as fixed and oxidation of methionine and acetylation of the N-terminus as variable modifications. For relative quantification the type "reporter ion MS2" was chosen and for all lysines and peptide N-termini TMT labels were defined. The mass accuracy for full mass spectra was set to 20 ppm (first search) and 4.5 ppm (second search), respectively and for MS/MS spectra to 20 ppm. The false discovery rates for peptide and protein identification were set to 1%. Only proteins for which at least two peptides were quantified were chosen for further validation. Relative quantification of proteins was carried out using the reporter ion MS2 algorithm implemented in MaxQuant.

The proteinGroups.txt file was used for further analysis. All proteins which were not identified with at least two razor and unique peptides in at least one biological replicate were filtered off. For further data analysis the "Reporter intensity corrected" corresponding to compound treatment was divided by the "Reporter intensity corrected" of the corresponding vehicle control and the results were written into a new column. This file was stored under a different file name in txt-format. For further data analysis Perseus version 1.6.2.3 was used.[6] The calculated ratios of the above-mentioned file were defined as main columns. Proteins resulting from the reverse database search, just identified by site, typical contaminants and not quantified in at least three out of three or four replicates, respectively, were filtered off. The ratios of the "Reporter intensities corrected" were logarithmized (log2) and normalized to the median. The mean of the replicates was calculated and the outlier test "Significance A" was performed. Proteins with a p-value < 0.00001 were considered as statistically highly significantly up- or down-regulated, depending on the

direction of change. Proteins that were up- or downregulated, respectively, by other, PDEδ-unrelated Pomalidomide-based PROTACs were removed from the lists. For graphical representation the p-value was logarithmized (-log10) and plotted against the normalized log2-value of the mean of reporter ion intensity corrected of the compound divided by the mean of the reporter ion intensity corrected of the DMSO vehicle control.

Cloning, expression and purification of recombinant proteins

The gene encoding Cereblon319-442 was PCR amplified from pAJ075_hsCRBN vector. pAJ075_hsCRBN vector was a gift from Eric Fischer (Addgene plasmid #124214; http://n2t.net/addgene:124214; RRID:Addgene_124214)[7].

The insert was subcloned into the BamHI and SalI restriction sites of the pGEX-6p-2rbs vector for bacterial expression with a N-terminal, PreScission-cleavable GST tag. The plasmid was verified by DNA sequencing.

Cereblon319-442 was expressed in Escherichia coli OverExpress C41(DE3) (Lucigen, #60442-1) in Terrific Broth medium for ~19 h at 18 °C after induction with 0.2 mM IPTG in the presence of 50 µM zinc acetate. Cells were harvested at 4,000 x g for 15 min and lysed by sonication in buffer containing 50 mM Hepes, pH 8.0, 500 mM NaCl, 5% (v/v) glycerol, 1 mM TCEP, 0.1% (v/v) Triton X-100 and protease-inhibitor mix HP plus (Serva). The cleared lysate was purified by affinity chromatography on a GSTrap FF column (GE Healthcare) using an ÄKTAprime plus (GE Healthcare). The GST tag was cleaved with GST-PreScission protease on the column over night at 4 °C. Cereblon319-442 was further purified by size-exclusion chromatography on a HiLoad 16/600 Superdex 75 pg (GE Healthcare) in buffer containing 20 mM Hepes, pH 8.0, 250 mM NaCl, 5% (v/v) glycerol and 1 mM TCEP.

Computational Modelling

The model is based on the crystal structure of Deltasonamide with PDEδ (PDB: 5ML3). The pyrimidine of Deltasonamide was replaced manually with the phenyl ring of the PROTAC probe

and minimized using the OPLS3e force field as implemented in the Schrödinger Release 2019-1 software package. For the phenyl ring, six different orientations were minimized. In the energetically most favourable orientation the amide carbonyl oxygen forms a hydrogen bond to the backbone NH of Met118 replacing a water molecule located in this position in the crystal structure of Deltasonamide with PDEδ (5ML3). During each minimization, the atoms of the ligand and protein atoms within a shell of six Å around the ligand were allowed to relax whereas the remaining atoms of PDEδ were kept fixed. Water molecules in the crystal structure were removed prior to minimization (Software: Schrödinger Release 2019-1: MacroModel, Schrödinger, LLC, New York, NY, 2019).

Chemical Synthesis

General information

All reactions were carried out in heat dried glassware under argon atmosphere. All commercially available compounds were used as provided without further purifications. They were purchased from the companies Acros Organics, Alfa Aesar, Sigma Aldrich, TCI Europe, or Fisher. Dry solvents were purchased as laboratory grade and used without further purification (e.g. DCM, Acetonitrile, DMF and THF). Analytical thin-layer chromatography (TLC) was performed on Merck silica gel aluminium plates with F-254 indicator. Compounds were visualized by irradiation with UV light or potassium permanganate staining. Column chromatography was performed using silica gel Acros 60 A. Column chromatography was performed using silica gel Merck 60 (particle size 0.040-0.063 mm).

NMR were either measured on DRX400 (400 MHz), Bruker DRX500 (500 MHz), INOVA500 (500 MHz) and DRX600 (600 MHz) at 300 K using $CDCl_3$ or CH_3OH as solvent and internal standard. Multiplicities are indicated as: s (singlet), d (doublet), t (triplet), q (quartet), quin (quintet), m (multiplet) and dd (doublet of a doublet); and coupling constants (*J*) are given in Hertz (Hz).

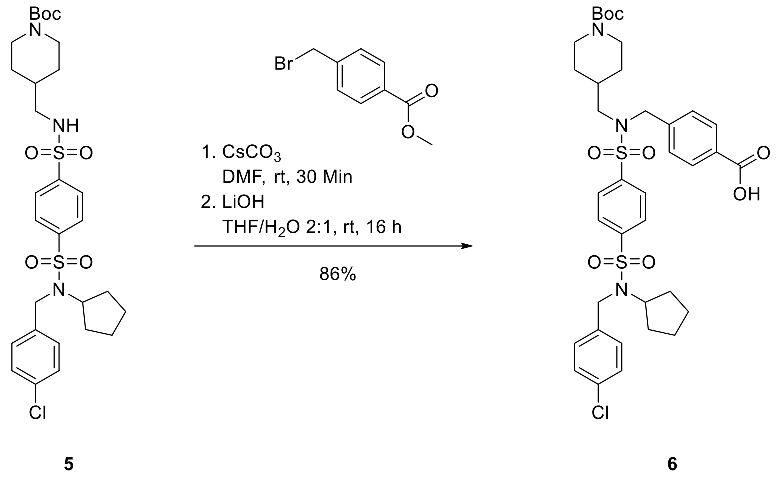

Figure S1: Synthesis of the PDEδ-binding part of the PROTACs.

tert-Butyl 4-(((4-(N-(4-chlorobenzyl)-N-cyclopentylsulfamoyl)phenyl)sulfonamido)methyl)-piperidine-1-carboxylate (5)

The compound was synthesized according to the literature.[8]

4-(((4-(N-(4-Chlorobenzyl)-N-cyclopentylsulfamoyl)-N-((1-pivaloylpiperidin-4-yl)methyl)phenyl)sulfonamido)methyl)benzoic acid (6)

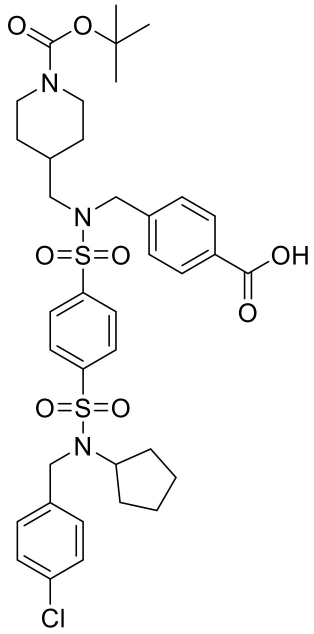

The amine **5** (2.1 g, 3.35 mmol, 1 eq.) and $CsCO_3$ (4.4 g, 13.41 mmol, 4 eq.) were dissolved in 40 ml DMF and after stirring for 30 min at room temperature methyl-4-(bromomethyl)benzoate (768 mg, 3.35 mmol, 1 eq.) was added. The reaction was quenched after 16 h with sat. NaCl solution and the crude product was extracted with ethyl acetate. After combining the organic phases, the solvent was removed under reduced pressure.

The ester (2.6 g, 3.35 mmol, 1 eq.) was used in the next reaction without any purification step in between. The compound was dissolved in 40 ml of a mixture of THF/H_2O (2:1) and LiOH (802 mg, 30 mmol,

10 eq.) was slowly added. After 16 h at room temperature the solvent was removed, and the residue was dissolved in cold acetonitrile. The suspension was filtered to afford the pure product (2.1 g, 2.88 mmol, 86%) as white solid.

^{1}H NMR (600 MHz, $CDCl_3$): δ = 8.05 (d, *J* = 8.3 Hz, 2H), 7.96 – 7.89 (m, 4H), 7.38 (d, *J* = 8.2 Hz, 2H), 7.33 – 7.28 (m, 5H), 4.38 (s, 2H, benzylic CH_2), 4.35 (s, 2H, benzylic CH_2), 4.34 – 4.28 (m, 1H, *c*-pen 1-H), 4.01 (d, *J* = 12.8 Hz, 2H, piperidine 2/6-H_B), 3.03 (d, *J* = 7.0 Hz, 2H, NH_2CH), 2.43 (t, *J* = 11.7 Hz, 2H, piperidine 2/6-H_A), 1.69 – 1.61 (m, 2H, *c*-pen 2/5-H_A), 1.58 – 1.49 (m, 4H, *c*-pen 3/4-H_B and piperidine 3/5-H_B), 1.49 – 1.44 (m, 3H, piperidine 4-H and *c*-pen 3/4-H_A), 1.42 (s, 9H, Boc), 1.34 – 1.23 (m, 2H, *c*-pen 2/5-H_B), 0.96 (qd, *J* = 12.6, 4.2 Hz, 2H, piperidine 3/5-H_A). **^{13}C NMR** (151 MHz, $CDCl_3$): δ = 169.34 (COOH), 154.83 (CO), 145.01 (Ar-C_q), 143.22 (Ar-C_q), 142.06 (Ar-C_q), 136.87 (Ar-C_q), 133.43 (Ar-C_q), 130.74 (CH-Ar), 129.17 (Ar-C_q), 128.85 (CH-Ar), 128.68 (CH-Ar), 128.47 (CH-Ar), 128.16 (CH-Ar), 128.03 (CH-Ar), 79.72 (C_q-Boc), 59.93 (*c*-Pen 1-C), 55.31 (N*C*H_2CH), 53.41 (benzylic CH_2), 47.16 (benzylic CH_2), 43.46 (piperidine 2/6-C), 35.27 (piperidine 4-C), 29.83 (piperidine 3/5-C), 29.59 (c-pen 2/5-C), 28.56 (3 x CH_3), 23.41 (*c*-pen 3/4-C). **HRMS** (ESI): $C_{37}H_{47}O_8N_3ClS_2$ $[M+H]^+$: calculated: 760.2488, found: 760.2490.

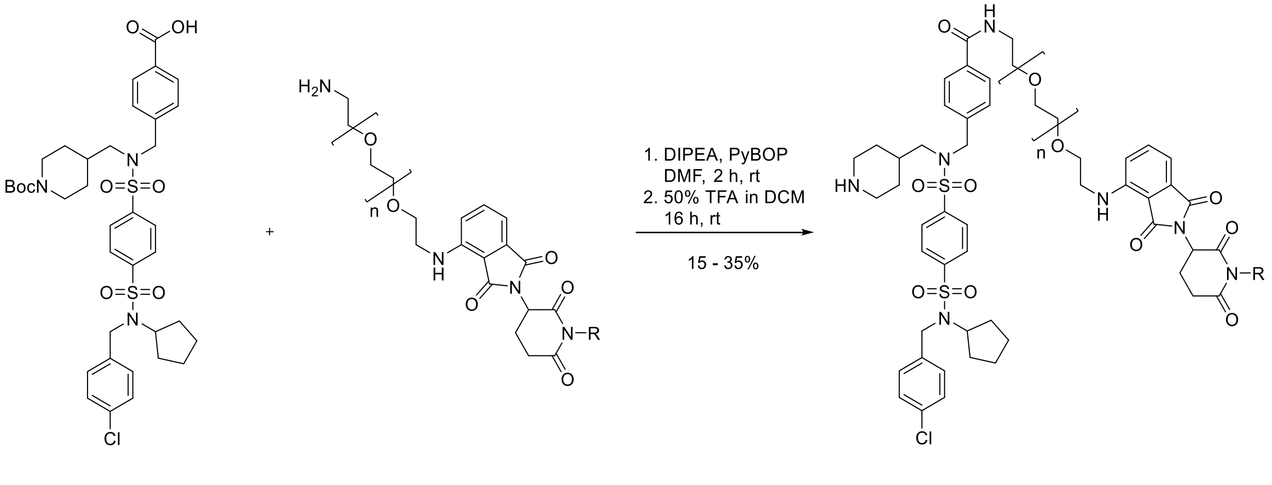

6

7: n = 1, R = H
8: n = 2, R = H
9: n = 3, R = H
10: n = 3, R = CH_3

1: n = 1, R = H
2: n = 2, R = H
3: n = 3, R = H, active PROTAC
4: n = 3, R = CH_3, inactive PROTAC

Figure S2: Last step of the synthesis to obtain heterobifunctional molecules.

Compounds 7-10

These compounds were synthesized according to literature.[9]

4-(((4-(N-(4-Chlorobenzyl)-N-cyclopentylsulfamoyl)-N-(piperidin-4-ylmethyl)phenyl)sulfonamido)methyl)-N-(14-((2-(1-methyl-2,6-dioxopiperidin-3-yl)-1,3-dioxoisoindolin-4-yl)amino)-3,6,9,12-tetraoxatetradecyl)benzamide (4)

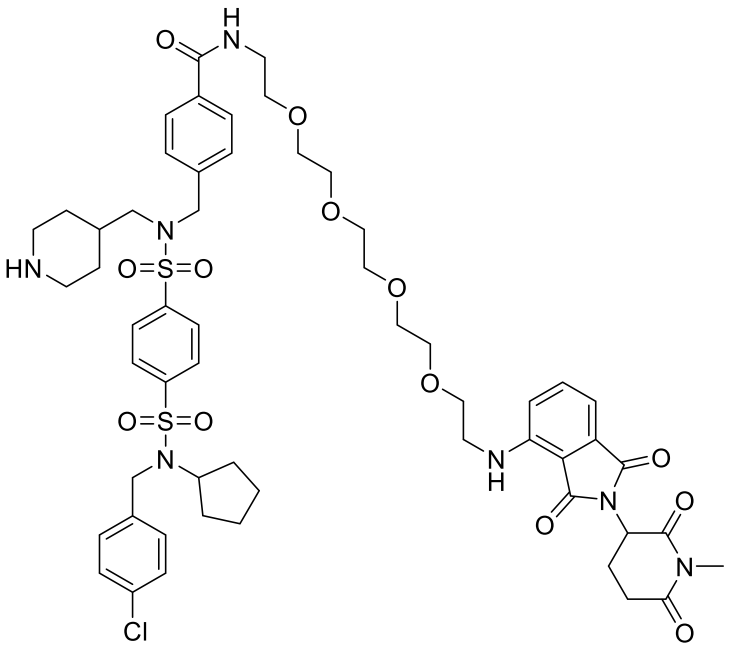

The carboxylic acid **6** (82.6 mg, 0.11 mmol, 1.1 eq.), DIPEA (14.0 mg, 0.1 mmol, 1 eq.) and PyBOP (56.5 mg, 0.11 mmol, 1.1 eq.) were dissolved in 2 ml DMF and stirred for 5 min at room temperature. Then the amine **16** (50 mg, 0.1 mmol, 1 eq.) was added under stirring and the mixture was left at room temperature for 2 h. The reaction was quenched with brine and the product was extracted with ethyl acetate. The organic phases were combined and dried over $MgSO_4$. After removing the solvent under reduced pressure, the product could be obtained by flash chromatography (silica, 5% methanol in dichloromethane) as a yellow oil.

The product was dissolved in 10 ml of 50% TFA in DCM and stirred for 30 min. The solvent was removed under a continuous air flow and the product was purified by means of reversed-phase HPLC (C_{18}, H_2O in acetonitrile 10 to 80% over 30 min) to yield 20.2 mg (0.02 mmol, 17%) of a yellow solid.

^{1}H NMR (700 MHz, CD_3OD): δ = 8.04 – 7.97 (m, 4H), 7.77 (d, *J* = 7.2 Hz, 2H), 7.53 (t, *J* = 7.7 Hz, 1H, , isoindoline 6-C), 7.37 (t, *J* = 7.0 Hz, 4H), 7.32 (d, *J* = 7.6 Hz, 2H), 7.06 (d, *J* = 8.5 Hz, 1H, isoindoline 5-H), 7.04 (d, *J* = 6.8 Hz, 1H, isoindoline 7-H), 5.07 (dd, *J* = 12.9 Hz, 5.2, 1H, dioxopiperidine 3-H), 4.42 (s, 2H, benzylic CH_2), 4.40 (s, 2H, benzylic CH_2), 4.36 – 4.32 (m, 1H,

c-Pen 1-H), 3.69 (t, *J* = 5.1 Hz, 2H, OCH_2), 3.65 – 3.59 (m, 14H, 7 × OCH_2), 3.55 (t, *J* = 5.1 Hz, 2H, NHCH_2), 3.47 (t, *J* = 5.2 Hz, 2H, NHCH_2), 3.30 – 3.28 (m, 2H, piperidine 2-H_A/6-H_A), 3.15 (d, *J* = 8.0 Hz, 2H, NCH_2CH), 3.11 (s, 3H, NCH_3), 2.89 – 2.85 (m, 1H, dioxopiperidine 4-H_A), 2.77 – 2.64 (m, 4H, piperidine 2-H_B/6-H_B and dioxopiperidine 5-H), 2.10 – 2.06 (m, 1H, dioxopiperidine 4-H_B), 1.80 (d, *J* = 13.0 Hz, 2H, piperidine 3-H_A/5-H_A), 1.71 – 1.66 (m, 1H, piperidine 4-H), 1.63 – 1.58 (m, 2H, *c*-pen 2-H/5-H), 1.57 – 1.52 (m, 2H, *c*-pen 3-H/4-H), 1.51 – 1.38 (2H, *c*-pen 3-H/4-H), 1.37 – 1.21 (m, 4H, *c*-pen 2-H/5-H and piperidine 3-H_B/5-H_B). **^{13}C NMR** (175 MHz, CD_3OD): δ = 173.66 (CO), 171.42 (CO), 170.71 (CO), 169.54 (CO), 169.30 (CO), 148.23 (Ar-C_q), 146.12 (Ar-C_q), 144.35 (Ar-C_q), 141.44 (Ar-C_q), 139.22 (Ar-C_q), 137.27 (isoindoline 6-C), 135.42 (Ar-C_q), 134.03 (Ar-C_q), 133.85 (Ar-C_q), 130.02 (Ar-CH), 129.82 (Ar-CH), 129.48 (Ar-CH), 129.38 (Ar-CH), 129.30 (Ar-CH), 128.74(Ar-CH), 118.34 (isoindoline 5-C), 112.07 (isoindoline 7-C), 111.25 (Ar-C_q), 71.61 (OCH_2), 71.57 (OCH_2), 71.56 (2 × OCH_2), 71.54 (OCH_2), 71.27 (OCH_2), 70.63 (OCH_2), 70.49 (OCH_2), 61.19 (*c*-Pen 1-C), 61.01 (CH), 55.31 (N*C*H_2CH), 53.85 (benzylic CH_2), 50.86 (dioxopiperidine 3-C), 47.97 (benzylic CH_2), 44.71 (piperidine 2-C), 43.25 ($NHCH_2$ [linker]), 41.05 ($NHCH_2$ [linker]), 33.86 (piperidine 4-C), 32.51(dioxopiperidine 5-C), 30.36 (*c*-pen 2-C/5-C), 27.54 (piperidine 3-C/5-C), 27.40 (NCH_3), 24.34 (*c*-pen 3-C/4-C), 23.05 (dioxopiperidine 4-C). **HRMS** (ESI): $C_{55}H_{68}O_{13}N_7ClNaS_2$ $[M+Na]^+$; calculated: 1156.3897, found: 1156.3905.

4-(((4-(N-(4-Chlorobenzyl)-N-cyclopentylsulfamoyl)-N-(piperidin-4-ylmethyl)phenyl)-sulfonamido)methyl)-N-(14-((2-(2,6-dioxopiperidin-3-yl)-1,3-dioxoisoindolin-4-yl)amino)-3,6,9,12-tetraoxatetradecyl)benzamide (3)

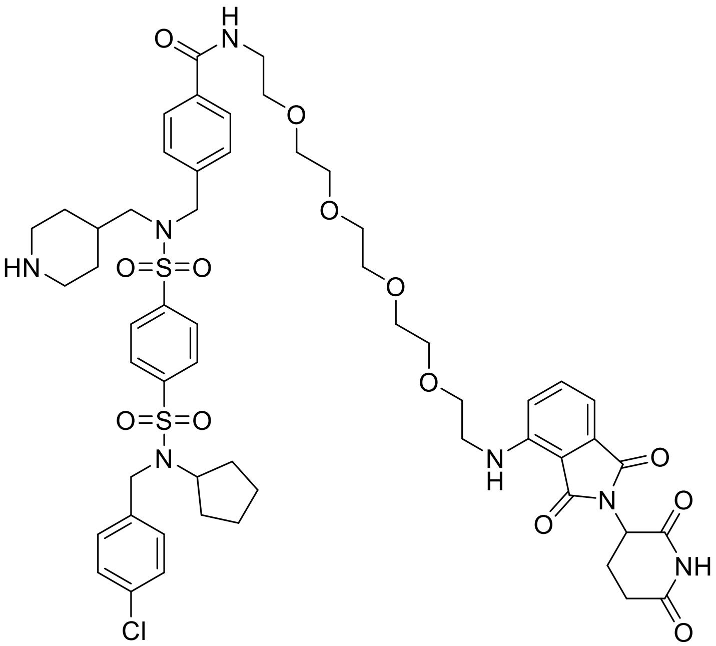

The carboxylic acid **6** (100.0 mg, 0.13 mmol, 1 eq.), DIPEA (24.6 µl, 144.7 µl, 1.1 eq.) and PyBOP (75.3 mg, 0.14 mmol, 1.1 eq.) were dissolved in 2 ml DMF and stirred for 5 min at room temperature. Then the amine **13** (121.8 mg, 0.25 mmol, 1.88 eq.) was added under stirring and left at room temperature for 2 h. Afterwards the reaction was quenched with brine and the product extracted with ethyl acetate. The organic phases were combined and dried over $MgSO_4$. After removing the solvent under reduced pressure, the product could be obtained by flash chromatography (silica, 5% methanol in dichloromethane) as a yellow oil.

The product was dissolved in 10 ml of 50% TFA in DCM and left stirring for 30 min. The solvent was removed under a continuous air flow and purified using reversed-phase HPLC (C_{18}, H_2O in acetonitrile 10 to 80% over 30 min). The product (52 mg, 50 µmol, 35%) could be obtained as yellow solid.

^{1}H NMR (700 MHz, CD_3OD): δ = 8.07 – 8.00 (m, 4H), 7.80 (d, *J* = 7.2 Hz, 2H), 7.56 (app. t, *J* = 7.6 Hz, 1H, isoindoline 6-C), 7.44 – 7.38 (m, 4H), 7.35 (d, *J* = 7.3 Hz, 2H), 7.09 (d, *J* = 8.5 Hz, 1H, isoindoline 5-H), 7.07 (d, *J* = 7.0 Hz, 1H, isoindoline 7-H), 5.07 (dd, *J* = 13.3, 5.6 Hz, 1H, dioxopiperidine 3-H), 4.45 (s, 2H, benzylic CH_2), 4.43 (s, 2H, benzylic CH_2), 4.40 – 4.34 (m, 1H, *c*-pen 1-H), 3.72 (t, *J* = 5.0 Hz, 2H, OCH_2), 3.68 – 3.62 (m, 14H, 7 × OCH_2), 3.58 (t, *J* = 5.1 Hz, 2H, $NHCH_2$), 3.50 (t, *J* = 5.0 Hz, 2H, $NHCH_2$), 3.32 – 3.30 (m, 2H, piperidine 2-H_A/6-H_A), 3.18 (d,

J = 7.3 Hz, 2H, NCH_2CH), 2.91 – 2.83 (m, 1H, dioxopiperidine 4-H_A), 2.80 – 2.68 (m, 4H, piperidine 2-H_B/6-H_B and dioxopiperidine 5-H), 2.15 – 2.10 (m, 1H, dioxopiperidine 4-H_B), 1.83 (d, J = 13.7 Hz, 2H, piperidine 3-H_A/5-H_A), 1.74 – 1.67 (m, 1H, piperidine 4-H), 1.67 – 1.61 (m, 2H, *c*-pen 3-H/4-H), 1.61 – 1.54 (m, 2H, *c*-pen 2-H/5-H), 1.50 – 1.45 (m, 2H, *c*-pen 3-H/4-H), 1.35 – 1.22 (m, 4H, *c*-pen 2-H/5-H and piperidine 3-H_B/5-H_B). **^{13}C NMR** (175 MHz, CD_3OD): δ = 174.63 (CO), 171.61 (CO), 170.69 (CO), 169.56 (CO), 169.28 (CO), 148.21 (Ar-C_q), 146.13 (Ar-C_q), 144.35 (Ar-C_q), 141.44 (Ar-C_q), 139.21 (Ar-C_q), 137.26 (isoindoline 6-C), 135.42 (Ar-C_q), 134.03 (Ar-C_q), 133.87 (Ar-C_q), 130.03 (Ar-CH), 129.83 (Ar-CH), 129.48 (Ar-CH), 129.37 (Ar-CH), 129.30 (Ar-CH), 128.75 (Ar-CH), 118.33 (isoindoline 5-C), 112.06 (isoindoline 7-C), 111.28 (Ar-C_q), 71.63 (OCH_2), 71.58 (OCH_2), 71.56 (2 × OCH_2), 71.53 (OCH_2), 71.27 (OCH_2), 70.60 (OCH_2), 70.49 (OCH_2), 61.20 (dioxopiperidine 3-C), 55.31 (N*C*H_2CH), 53.86 (benzylic CH_2), 50.21 (N*C*H(*c*-pen)), 47.98 (benzylic CH_2), 44.72 (piperidine 2-C), 43.25 (NHCH_2 [linker]), 41.04 (NHCH_2 [linker]), 33.85 (piperidine 4-C), 32.21 (CH_2), 30.36 (CH_2), 27.54 (CH_2), 24.34 (CH_2), 23.81 (CH_2). **HRMS** (ESI): $C_{55}H_{68}O_{13}N_7ClNaS_2$ $[M+Na]^+$; calculated: 1156.3897, found: 1156.3905.

4-(((4-(N-(4-Chlorobenzyl)-N-cyclopentylsulfamoyl)-N-(piperidin-4-ylmethyl)phenyl)sulfonamido)methyl)-N-(2-(2-(2-(2-((2-(2,6-dioxopiperidin-3-yl)-1,3-dioxoisoindolin-4-yl)amino)ethoxy)ethoxy)ethoxy)ethyl)benzamide (2)

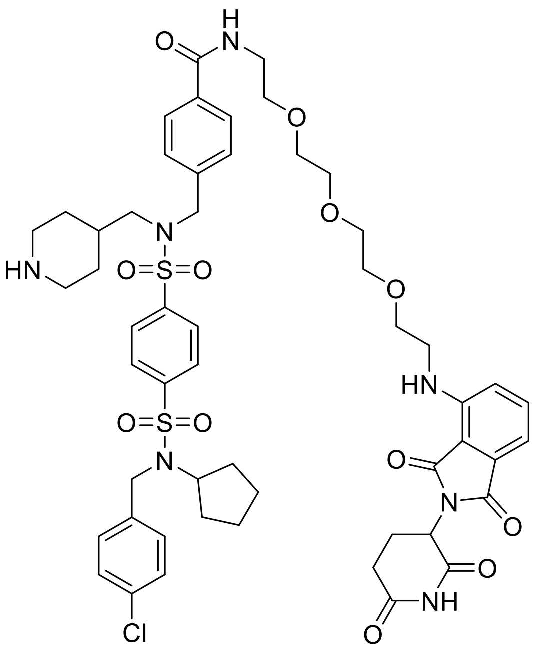

The carboxylic acid **6** (100 mg, 0.13 mmol, 1 eq.), PyBOP (75.3 mg, 0.14 mmol, 1.1 eq.) and DIPEA (24.6 µl, 0.14 mmol, 1.1 eq.) were dissolved in 2 ml DMF and stirred for 5 min at room temperature. Then the amine **12** (110.9 mg, 0.25 mmol, 1.88 eq.) was added under stirring and left at room temperature for 2 h. Afterwards the reaction was quenched with brine and the product extracted with ethyl acetate. The organic phases were combined and dried over $MgSO_4$. After removing the solvent under reduced pressure, the product could be obtained by flash chromatography (silica, 5% methanol in dichloromethane) as a yellow oil.

The product was dissolved in 10 ml of 50% TFA in DCM and left stirring for 30 min. The solvent was removed under a continuous air flow and purified using reversed-phase HPLC (C_{18}, H_2O in acetonitrile 10 to 80% over 30 min). The product (41.9 mg, 0.04 mmol, 29%) could be obtained as yellow solid.

^{1}H NMR (700 MHz, CD_3OD): δ = 8.04 – 7.97 (m, 4H), 7.75 (d, *J* = 8.4 Hz, 2H), 7.53 (dd, *J* = 8.5, 7.1 Hz, 1H), 7.38 (d, *J* = 8.6 Hz, 2H), 7.35 (d, *J* = 8.3 Hz, 2H), 7.32 (d, *J* = 8.5 Hz, 2H), 7.05 (d, *J* = 8.5 Hz, 1H), 7.04 (d, *J* = 7.0 Hz, 1H), 5.04 (dd, *J* = 12.8, 5.5 Hz, 1H, dioxopiperidine), 4.43 (s, 2H), 4.38 (s, 2H), 4.34 (m, 1H, *c*-pen 1-H), 3.68 (t, *J* = 5.3 Hz, 2H), 3.67 – 3.62 (m, 10H), 3.55 (t, *J* = 5.4 Hz, 2H), 3.45 (t, *J* = 5.3 Hz, 2H), 3.29 – 3.27 (m, 2H, piperidine 2-H_A/6-H_A), 3.15 (d, *J* = 7.4 Hz, 2H, N*H_2*CH), 2.88 – 2.82 (m, 1H, dioxopiperidine 4-H_B), 2.77 – 2.66 (m, 3H, piperidine 2-H_A/6-H_A and dioxopiperidine 4-H_A), 2.14 – 2.07 (m, 1H, dioxopiperidine 5-H_A), 1.81 (d, *J* = 12.0 Hz, 2H,

piperidine 3-H_A/5-H_A), 1.70 – 1.65 (m, 1H, piperidine 4-H), 1.64 – 1.59 (m, 2H, *c*-pen), 1.58 – 1.53 (m, 2H, *c*-pen), 1.48 – 1.43 (m, 2H, *c*-pen), 1.36 – 1.28 (m, 3H, *c*-pen and dioxopiperidine 5-H_B), 1.24 (d, *J* = 11.2 Hz, 2H, piperidine 3-H_B/5-H_B). **^{13}C NMR** (175 MHz, CD_3OD): δ = 174.63 (CO), 171.64 (CO), 170.68 (CO), 169.56 (CO), 169.28 (CO), 148.19 (Ar-C_q), 146.17 (Ar-C_q), 144.37 (Ar-C_q), 141.41 (Ar-C_q), 139.21 (Ar-C_q), 137.25 (isoindoline 6-C), 135.44 (Ar-C_q), 134.05 (Ar-C_q), 133.88 (Ar-C_q), 130.03 (Ar-CH), 129.81 (Ar-CH), 129.48 (Ar-CH), 129.37 (Ar-CH), 129.29 (Ar-CH), 128.73 (Ar-CH), 118.30 (isoindoline 5-C), 112.04 (isoindoline 7-C), 111.29, 111.28 (Ar-C_q), 71.66 (OCH_2), 71.57 (2 × OCH_2), 71.32 (OCH_2), 70.59 (OCH_2), 70.48 (OCH_2), 61.21 (N*C*H(*c*-pen)), 55.27 (N*C*H_2CH), 53.81 (benzylic CH_2), 50.21 (dioxopiperidine 3-C), 47.98 (benzylic CH_2), 44.73 (piperidine 2-C), 43.23 (NHCH_2 [linker]), 41.05 (NHCH_2 [linker]), 33.84 (piperidine 4-C), 32.21 (CH_2), 30.36 (CH_2), 27.55 (CH_2), 26.91 (CH_2), 24.34 (CH_2), 23.82 (CH_2), 23.73 (CH_2). **HRMS** (ESI): $C_{53}H_{65}O_{12}N_7ClS_2$ $[M+H]^+$; calculated: 1090.3816, found: 1090.3825.

4-(((4-(N-(4-Chlorobenzyl)-N-cyclopentylsulfamoyl)-N-(piperidin-4-ylmethyl)phenyl)-sulfonamido)methyl)-N-(2-(2-(2-((2-(2,6-dioxopiperidin-3-yl)-1,3-dioxoisoindolin-4-yl)amino)ethoxy)ethoxy)ethyl)benzamide (1)

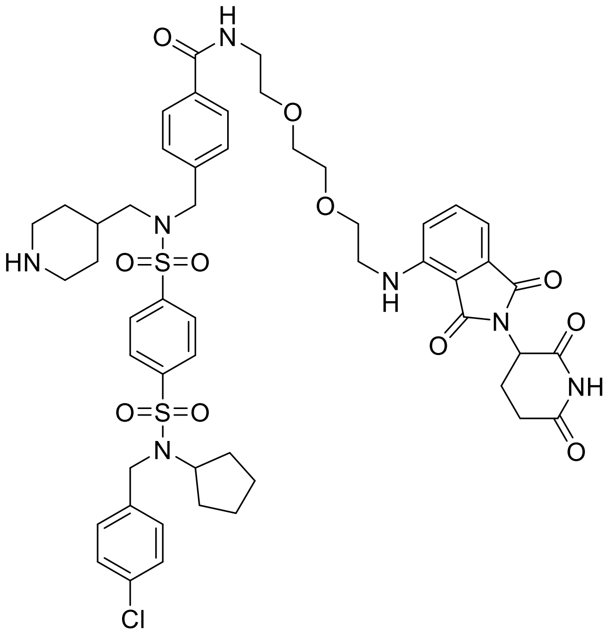

The carboxylic acid **6** (100 mg, 0.13 mmol, 1 eq.), PyBOP (75.3 mg, 0.14 mmol, 1.1 eq.) and DIPEA (22.4 ml, 0.13 mmol, 1.0 eq.) were dissolved in 2 ml DMF and stirred for 5 min at room temperature. Then, the amine **11** (100 mg, 0.25 mmol, 1.88 eq.) was added under stirring and left at room temperature for 2 h. Afterwards the reaction was quenched with brine and the product extracted with ethyl acetate. The organic phases were combined and dried over $MgSO_4$.

After removing the solvent under reduced pressure, the product could be obtained by flash chromatography (silica, 5% methanol in dichloromethane) as a yellow oil.

The product was dissolved in 10 ml of 50% TFA in DCM and left stirring for 30 min. The solvent was removed under a continuous air flow and purified using reversed-phase HPLC (C_{18}, H_2O in acetonitrile 10 to 80% over 30 min). The product (21.2 mg, 0.02 mmol, 15%) could be obtained as yellow solid.

HRMS (ESI): $C_{52}H_{61}O_{11}N_7ClS_2$ $[M+H]^+$; calculated: 1046.3554, found: 1046.3563.

Synthesis of VHL-based PDEδ PROTAC

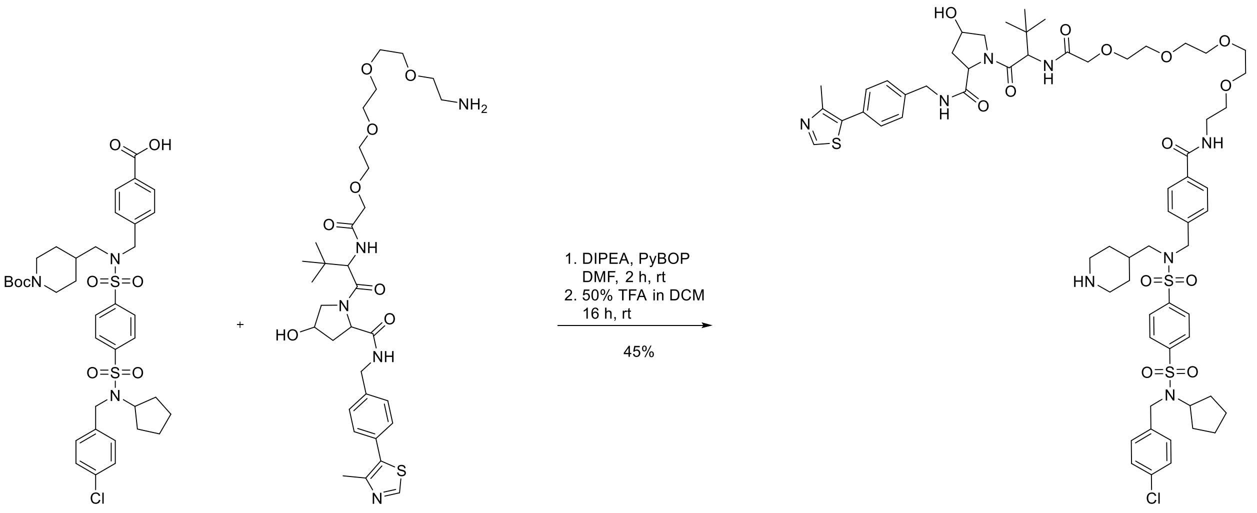

6

5

Figure S3: Synthesis of VHL-bases PDEδ PROTAC 5.

1-(18-(tert-butyl)-1-(4-(((4-(N-(4-chlorobenzyl)-N-cyclopentylsulfamoyl)-N-(piperidin-4-ylmethyl)phenyl)sulfonamido)methyl)phenyl)-1,16-dioxo-5,8,11,14-tetraoxa-2,17-diazanonadecan-19-oyl)-4-hydroxy-N-(4-(4-methylthiazol-5-yl)benzyl)pyrrolidine-2-carboxamide (5)

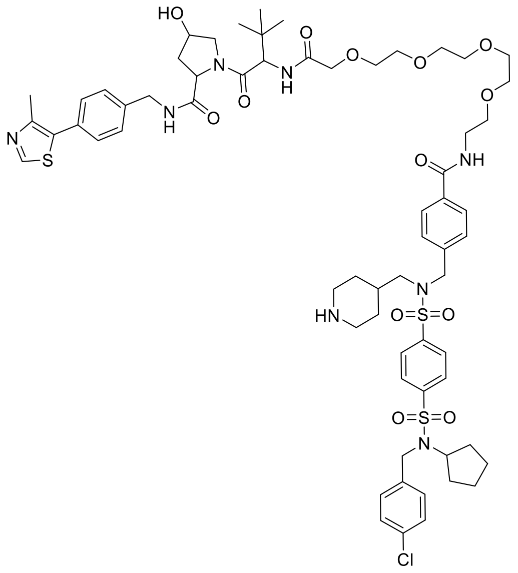

The carboxylic acid **6** (20 mg, 0.03 mmol, 1 eq.), PyBOP (15.1 mg, 0.03 mmol, 1.1 eq.) and DIPEA (7.1 ml, 0.03 mmol, 1 eq.) were dissolved in 2 ml DMF and stirred for 5 min at room temperature. Then, the amine (20.2 mg, 0.03 mmol, 1.1 eq.) was added under stirring and left at room temperature for 2 h. Afterwards the reaction was quenched with brine and the product extracted with ethyl acetate. The organic phases were combined and dried over $MgSO_4$. After removing the solvent under reduced pressure, the product could be obtained by flash chromatography (silica, 5% methanol in dichloromethane) as a yellow oil.

The product was dissolved in 10 ml of 50% TFA in DCM and left stirring for 30 min. The solvent was removed under a continuous air flow and purified using reversed-phase HPLC (C_{18}, H_2O in acetonitrile 10 to 80% over 30 min). The product (15 mg, 0.01 mmol, 45%) could be obtained as yellow solid.

HRMS (ESI): $C_{52}H_{61}O_{11}N_7ClS_2$ $[M+H]^+$; calculated: 1307.5130, found: 1307.5193.

References

[1] A. Fabregat, K. Sidiropoulos, G. Viteri, O. Forner, P. Marin-Garcia, V. Arnau, P. D'Eustachio, L. Stein, H. Hermjakob, *BMC Bioinf.* **2017**, *18*, 142.

[2] G. Zimmermann, B. Papke, S. Ismail, N. Vartak, A. Chandra, M. Hoffmann, S. A. Hahn, G. Triola, A. Wittinghofer, Bastiaens, Philippe I H et al., *Nature* **2013**, *497*, 638.

[3] K. A. Dooley, S. Millinder, T. F. Osborne, *J. Biol. Chem.* **1998**, *273*, 1349.

[4] M. Schmick, N. Vartak, B. Papke, M. Kovacevic, D. C. Truxius, L. Rossmannek, P. I. Bastiaens, *Cell* **2014**, *157*, 459.

[5] J. Cox, M. Mann, *Nat. Biotechnol.* **2008**, *26*, 1367.

[6] S. Tyanova, T. Temu, P. Sinitcyn, A. Carlson, M. Y. Hein, T. Geiger, M. Mann, J. Cox, *Nature methods* **2016**, *13*, 731.

[7] R. P. Nowak, S. L. DeAngelo, D. Buckley, Z. He, K. A. Donovan, J. An, N. Safaee, M. P. Jedrychowski, C. M. Ponthier, M. Ishoey et al., *Nat. Chem. Biol.* **2018**, *14*, 706.

[8] P. Martín-Gago, E. K. Fansa, C. H. Klein, S. Murarka, P. Janning, M. Schürmann, M. Metz, S. Ismail, C. Schultz-Fademrecht, M. Baumann et al., *Angew. Chem., Int. Ed. Engl.* **2017**, *56*, 2423.

[9] C. Steinebach, S. Lindner, N. D. Udeshi, D. C. Mani, H. Kehm, S. Köpff, S. A. Carr, M. Gütschow, J. Krönke, *ACS Chem. Biol.* **2018**, *13*, 2771.

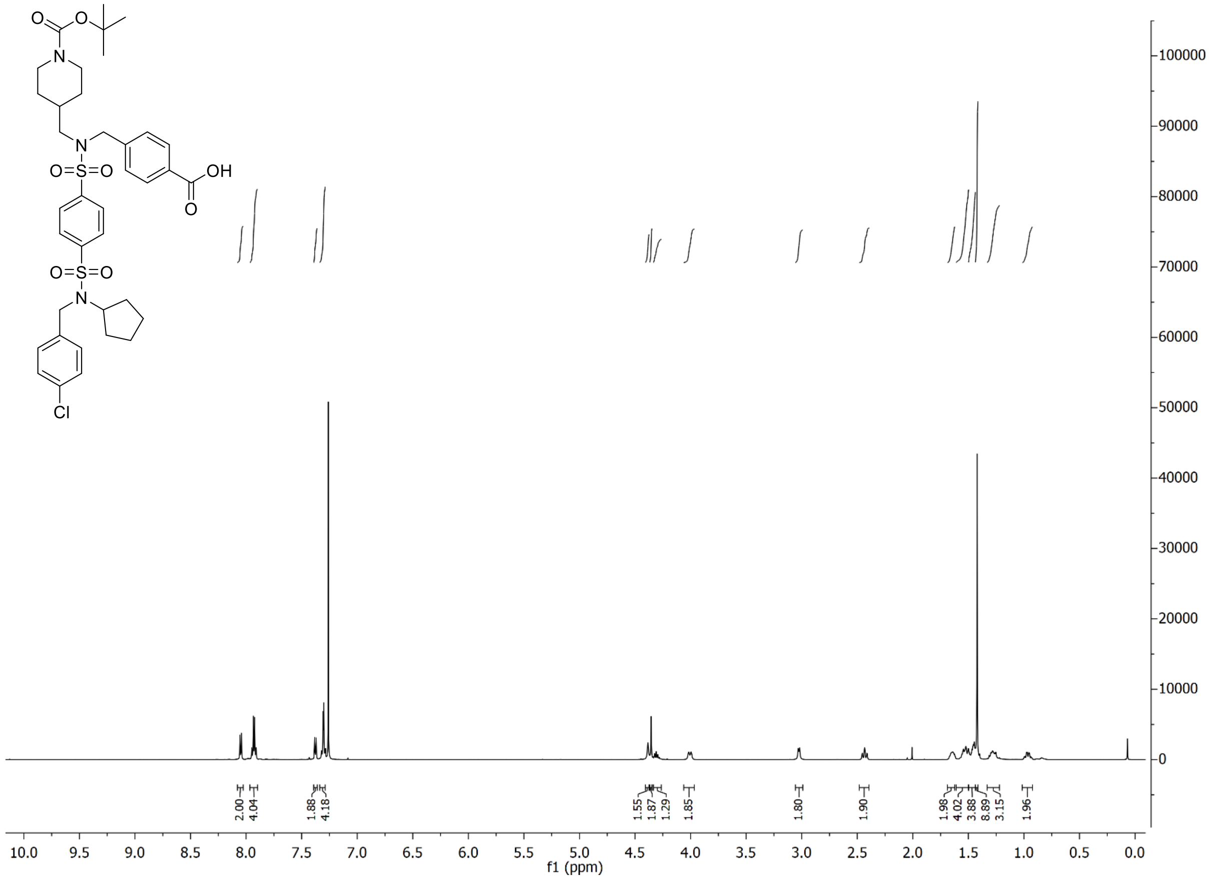
2.00
4.04
1.88
4.18
1.55
1.87
1.29
1.85
1.80
1.90
1.98
4.02
3.88
8.89
3.15
1.96
f1 (ppm)

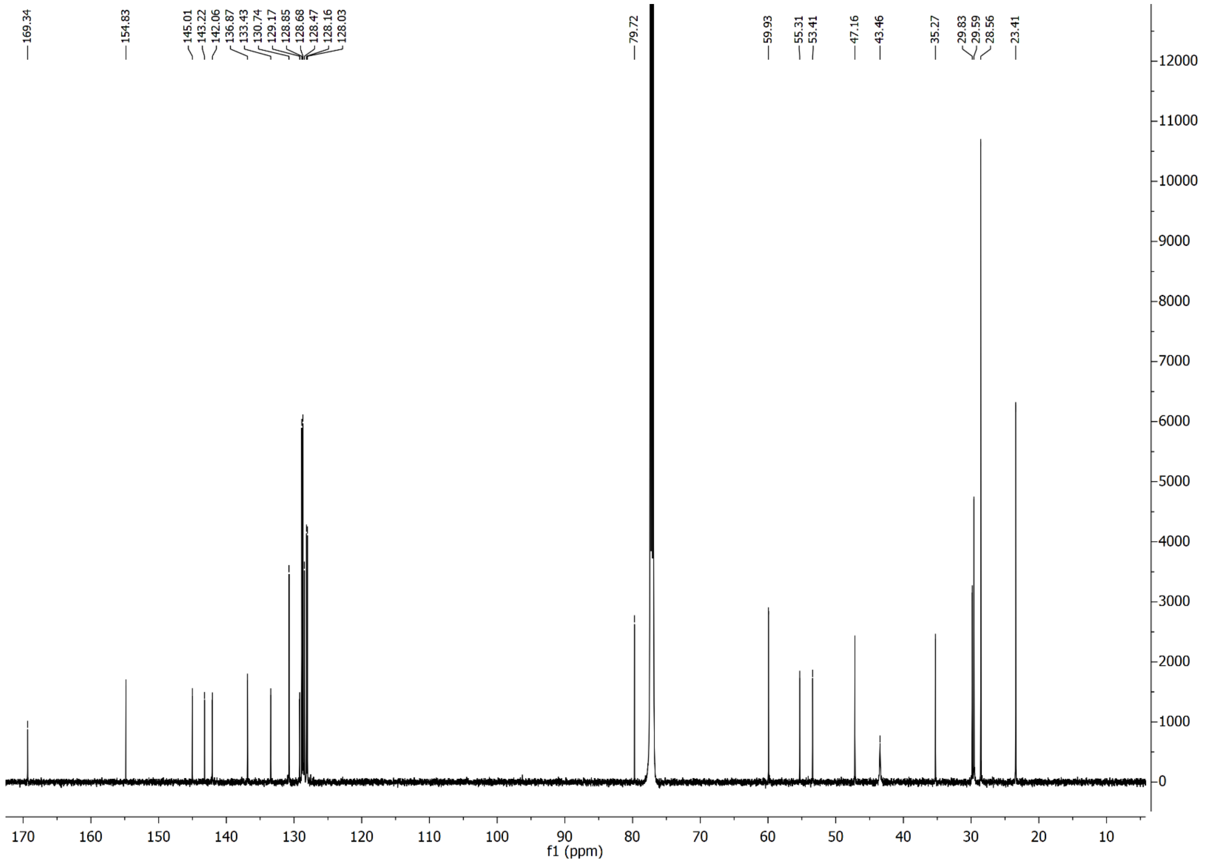
169.34
154.83
145.01
143.22
142.06
136.87
133.43
130.74
129.17
128.85
128.68
128.47
128.16
128.03
79.72
59.93
55.31
53.41
47.16
43.46
35.27
29.83
29.59
28.56
23.41
f1 (ppm)

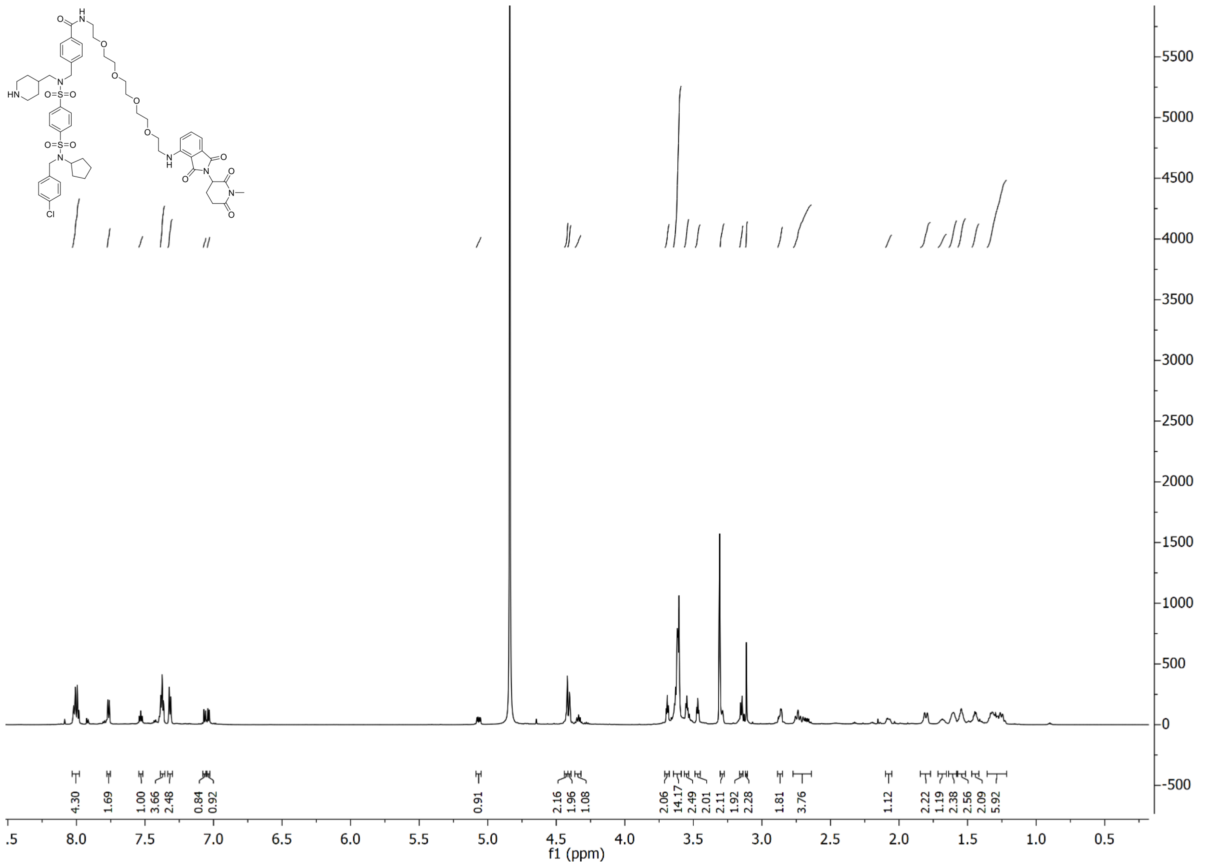

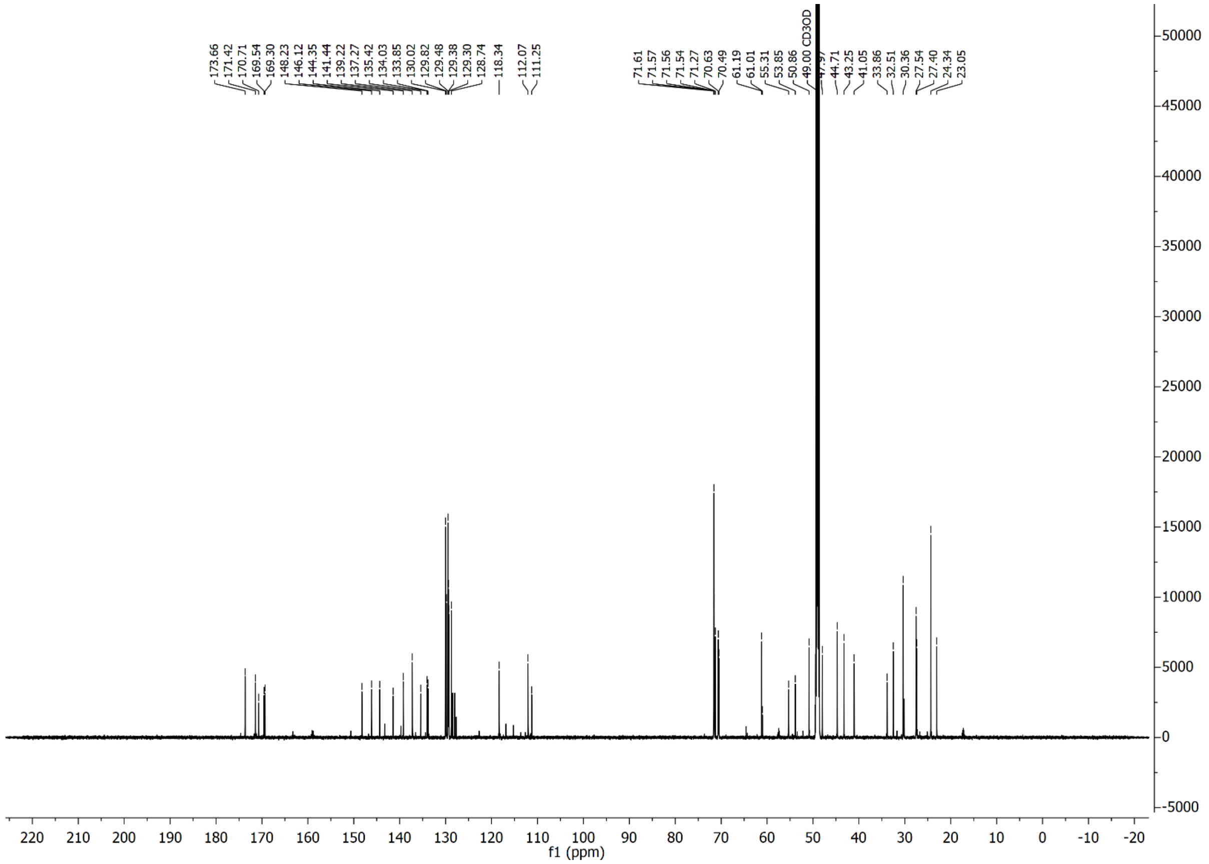
173.66
171.42
170.71
169.54
169.30
148.23
146.12
144.35
141.44
139.22
137.27
135.42
134.03
133.85
130.02
129.82
129.48
129.38
129.30
128.74
118.34
112.07
111.25
71.61
71.57
71.56
71.54
71.27
70.63
70.49
61.19
61.01
55.31
53.85
50.86
49.00 CD3OD
47.97
44.71
43.25
41.05
33.86
32.51
30.36
27.54
27.40
24.34
23.05
f1 (ppm)

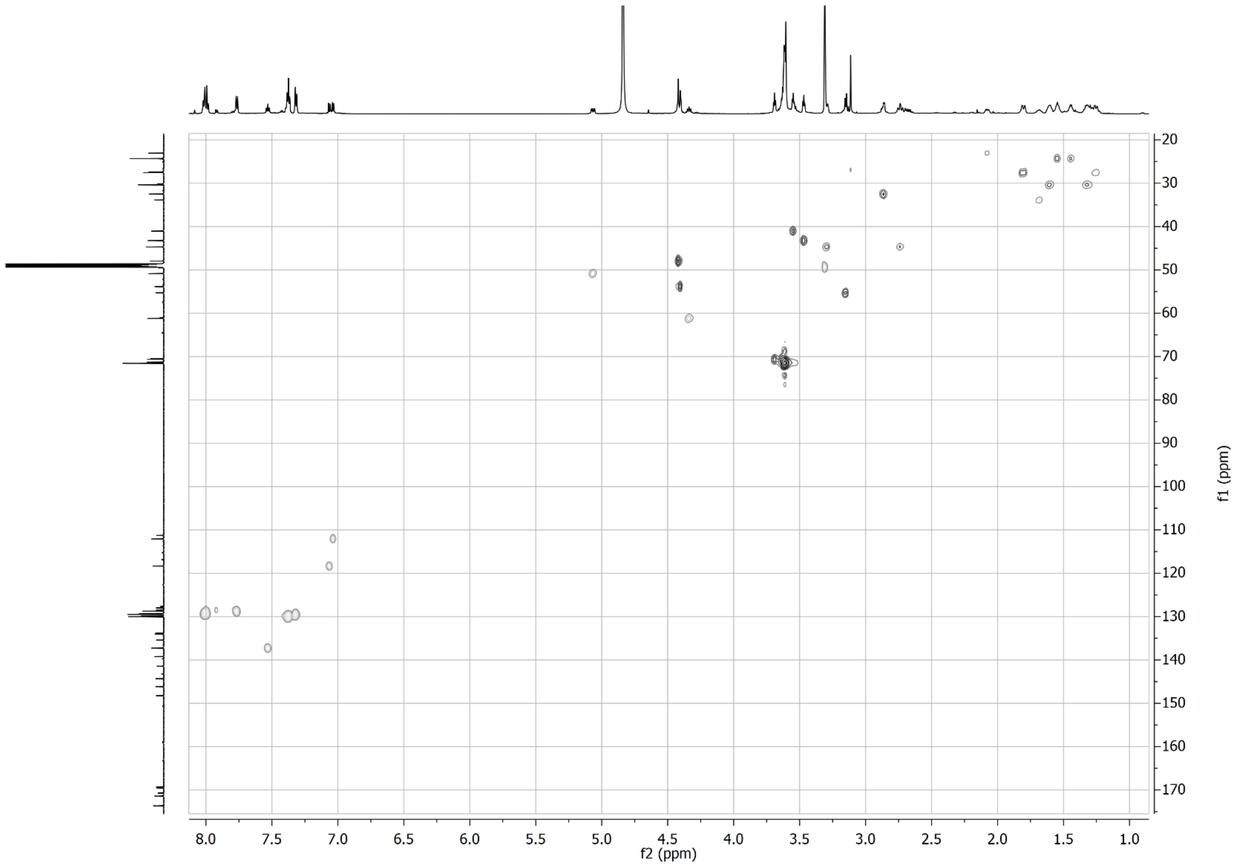
f2 (ppm)
f1 (ppm)
8.0 7.5 7.0 6.5 6.0 5.5 5.0 4.5 4.0 3.5 3.0 2.5 2.0 1.5 1.0
20 30 40 50 60 70 80 90 100 110 120 130 140 150 160 170

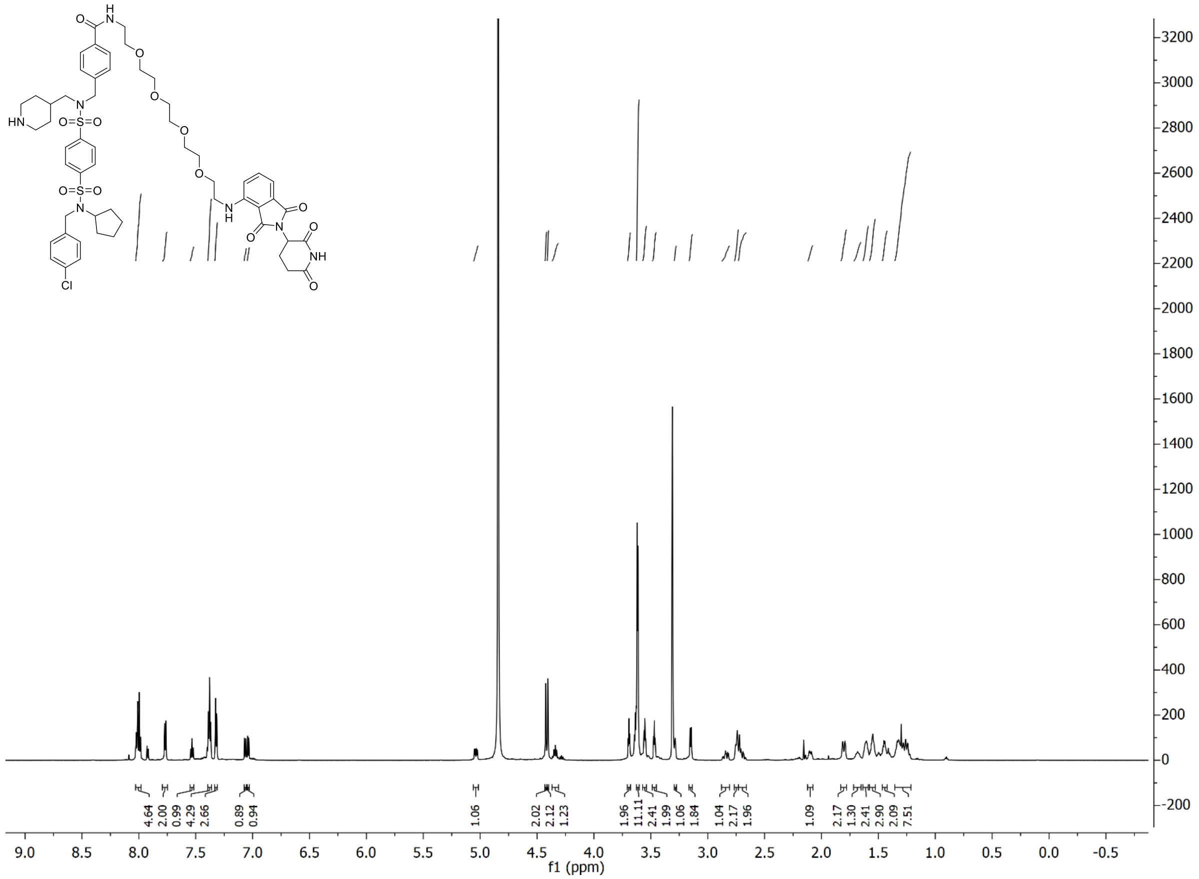
f1 (ppm)

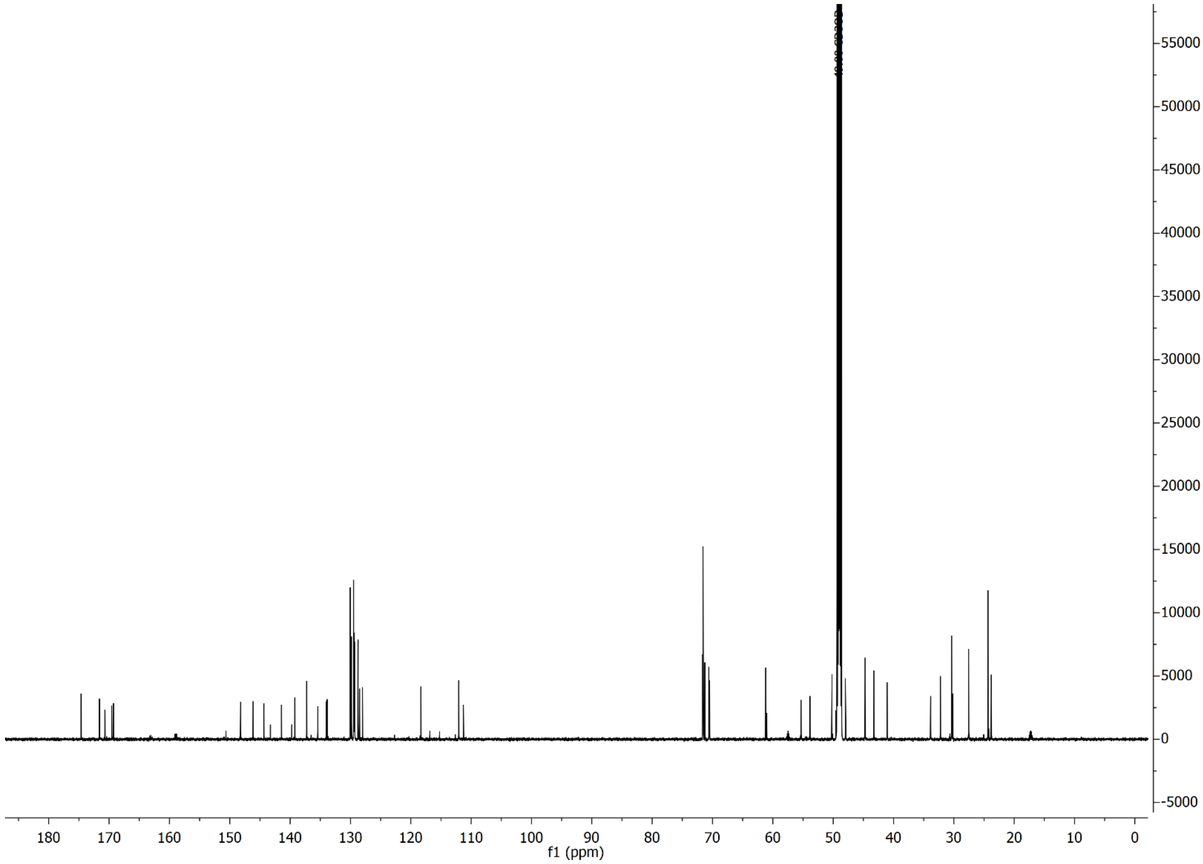
f1 (ppm)

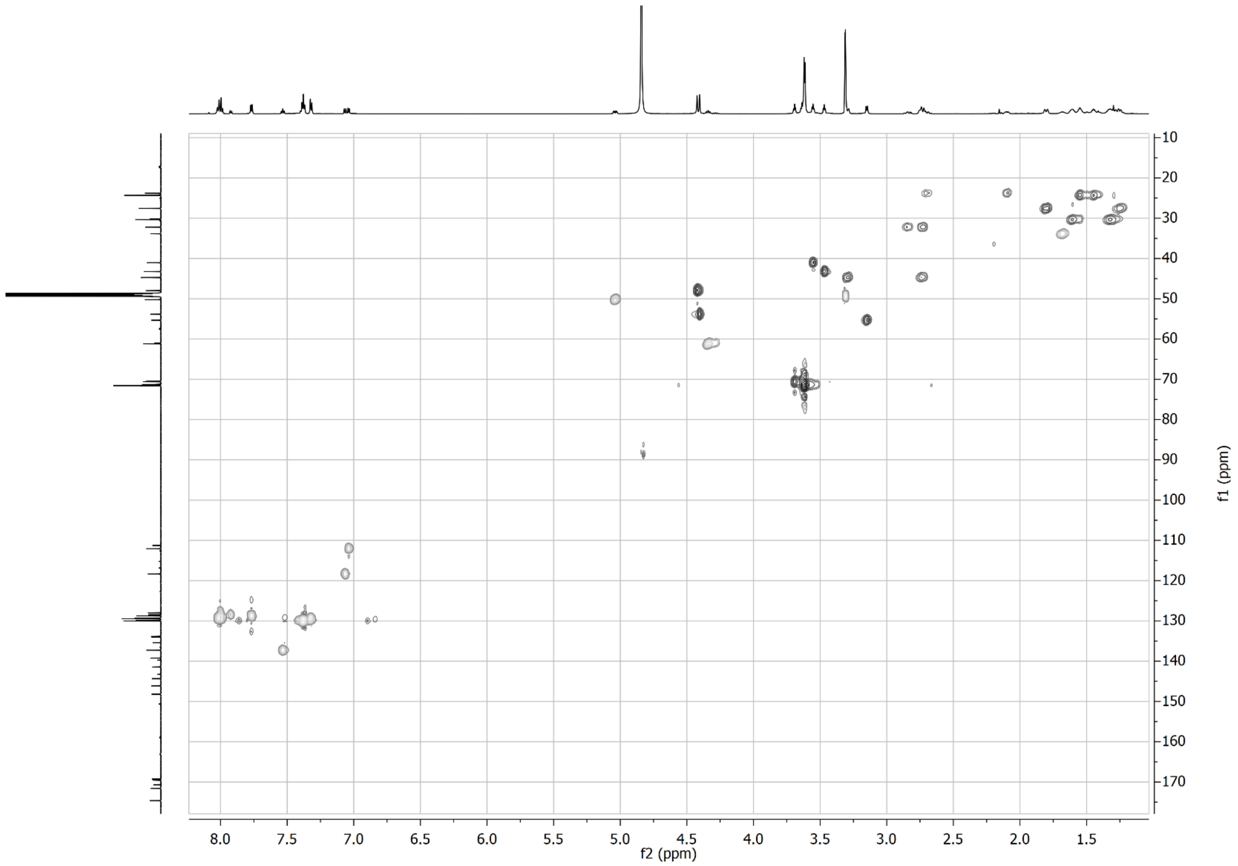

f2 (ppm)
f1 (ppm)

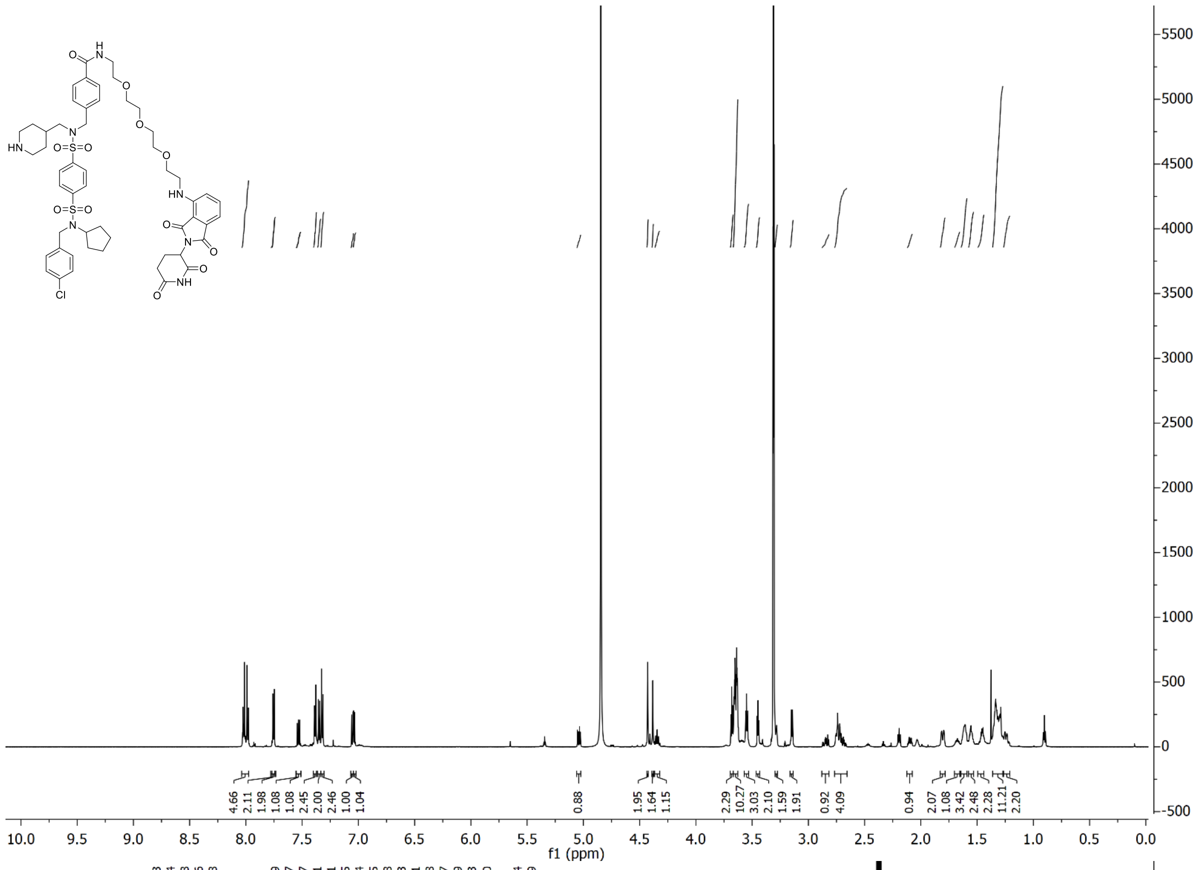

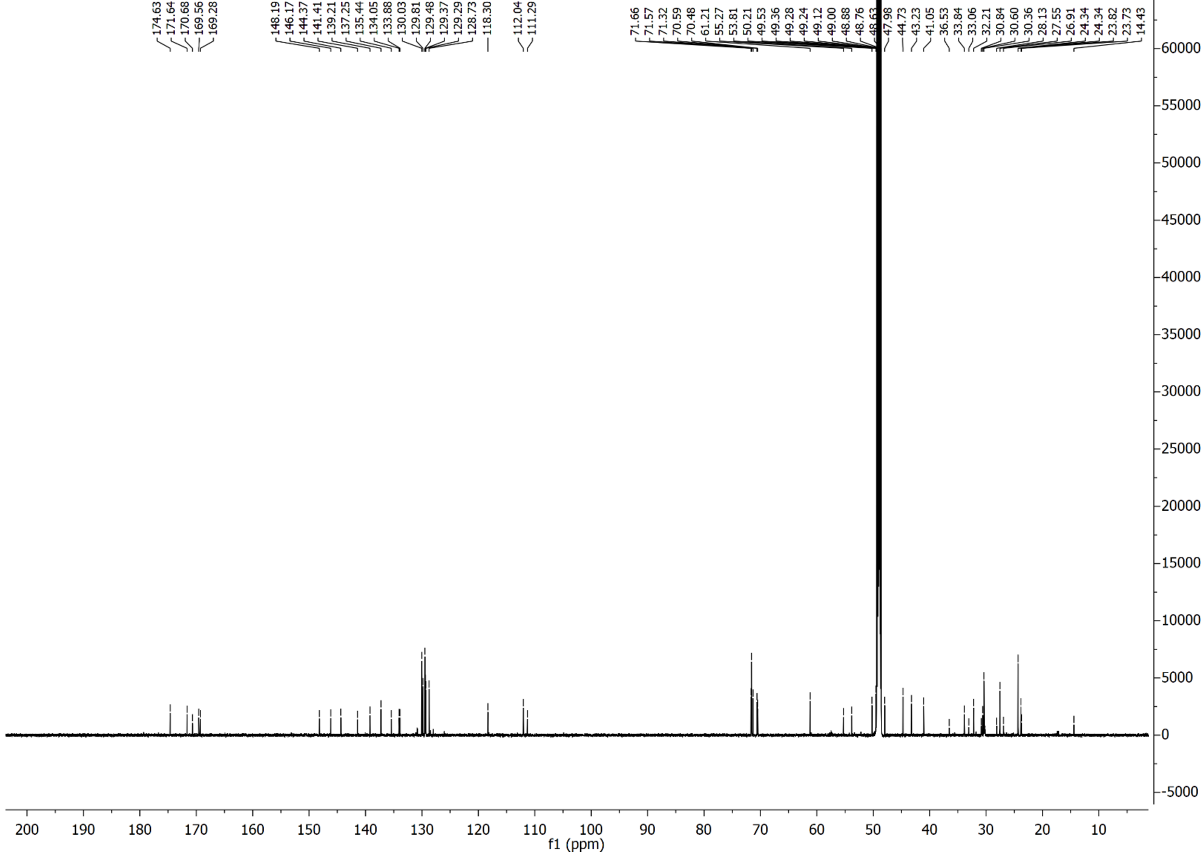

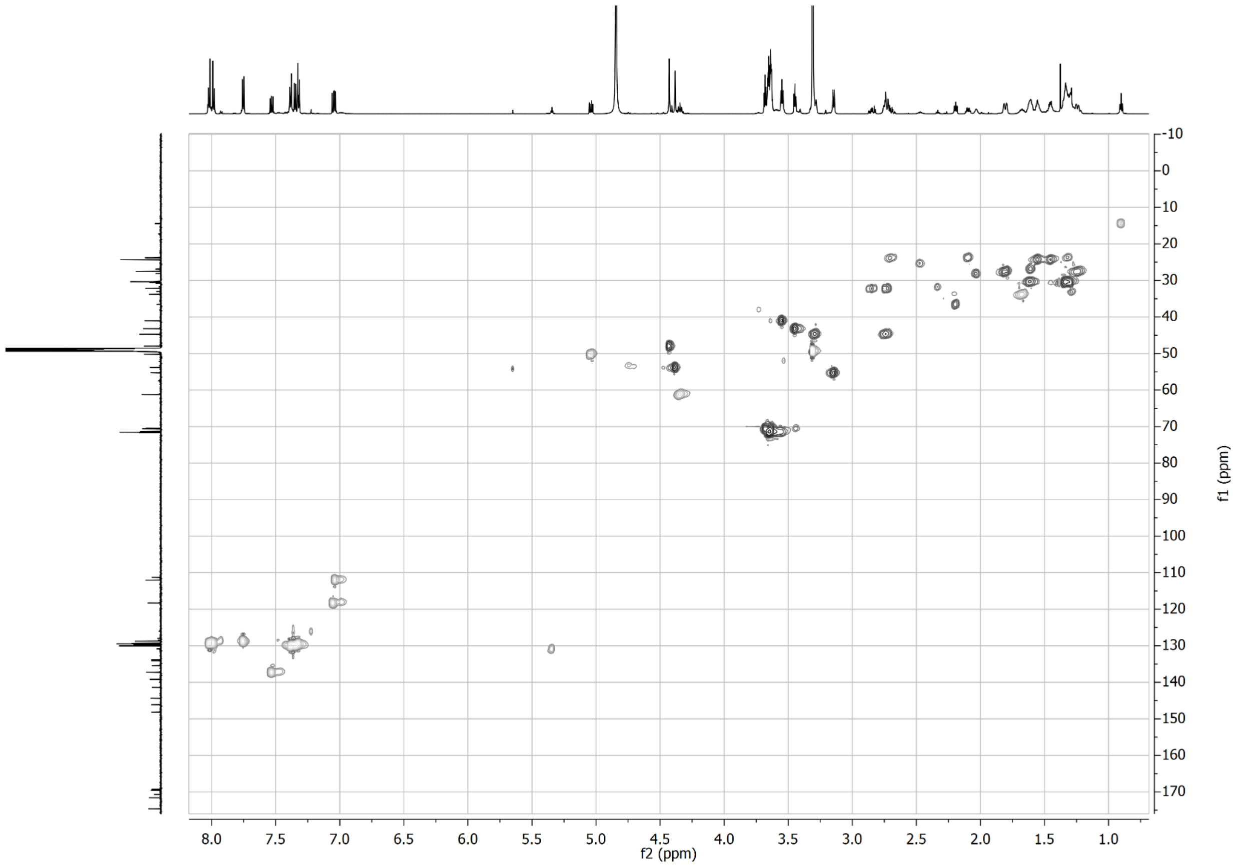

f2 (ppm)
f1 (ppm)

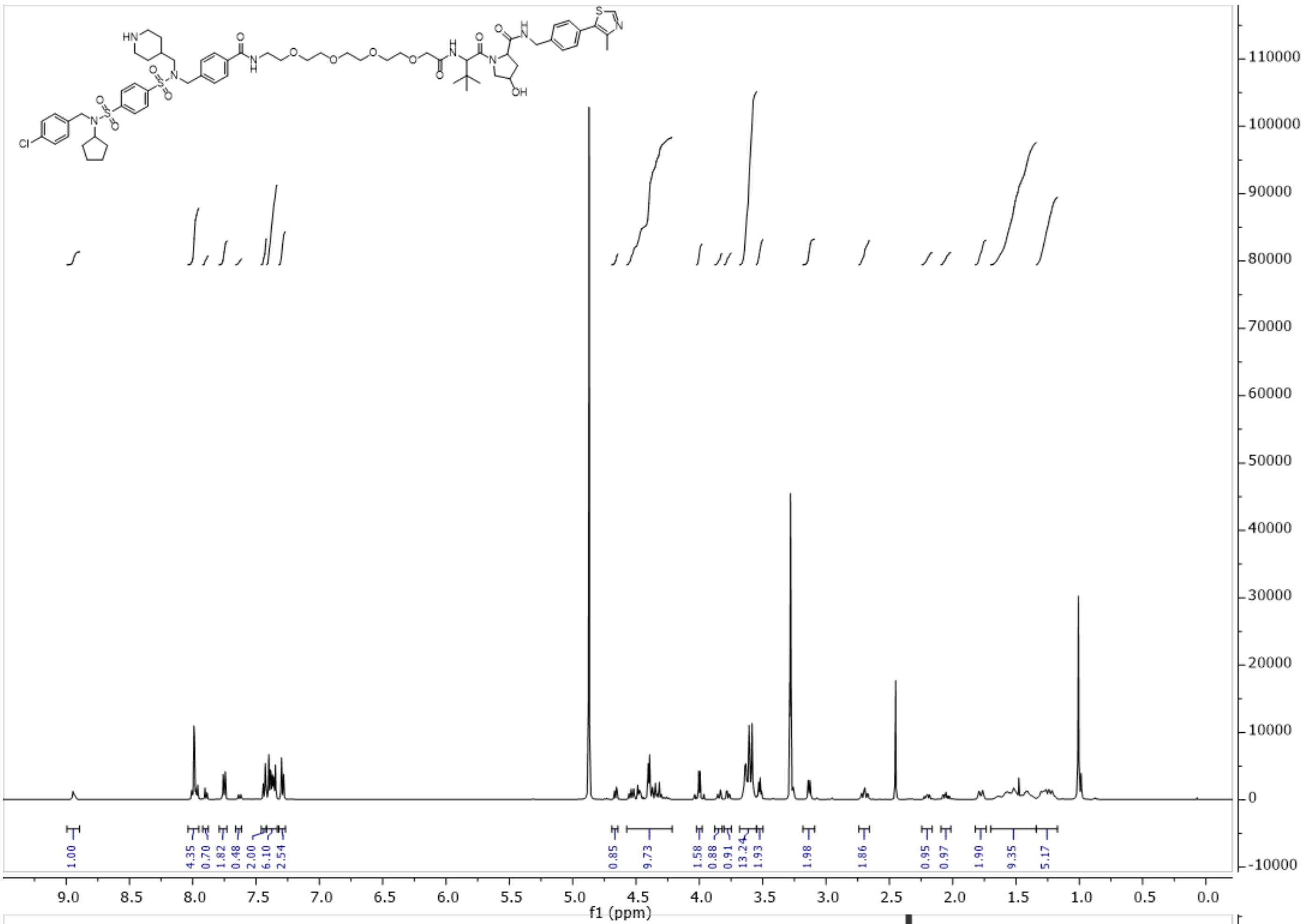
f1 (ppm)

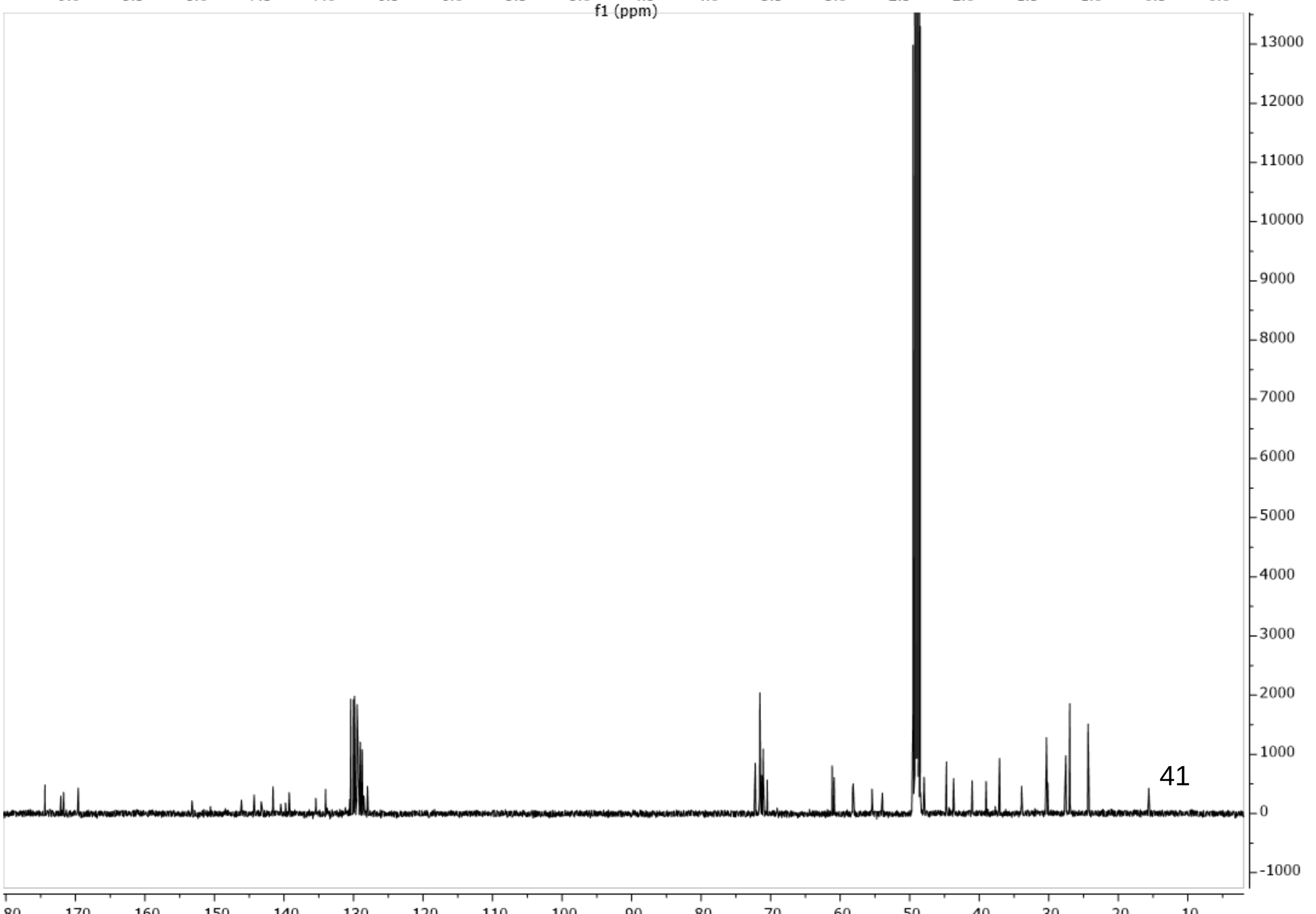

Supplement: Supplementary file 1 — Supplementary [file ANIE-59-5595-s001.pdf]
